# Jian Pi Hua Tan Fang Reverses Trastuzumab Resistance of HER2‐Positive Gastric Cancer Through PI3K/AKT/mTOR Pathway: Integrating Network Pharmacology, Molecular Docking and Experimental Validation

**DOI:** 10.1002/iid3.70154

**Published:** 2025-02-07

**Authors:** Jia Hu, Wenjing Bu, Yongfang Ding, Xin Li, Bo Zhang, Bo Shen, Cong Wu, Youqi Xu, Xiaoyang Zhang

**Affiliations:** ^1^ Nanjing Integrated Traditional Chinese and Western Medicine Hospital Affiliated with Nanjing University of Chinese Medicine Nanjing China; ^2^ The Second Affiliated Hospital of Nanjing University of Chinese Medicine Nanjing China; ^3^ Jiangsu Province Academy of Traditional Chinese Medicine Nanjing China

**Keywords:** HER2‐positive gastric cancer, Jian Pi Hua Tan Fang, molecular docking, network pharmacology, PI3K/AKT/mTOR pathway, Trastuzumab resistance

## Abstract

**Background:**

Currently, trastuzumab resistance significantly impacts the treatment outcome for individuals with HER2‐positive gastric cancer. In clinical practice, Jian Pi Hua Tan Fang (JPHTF) has been shown to be effective in preventing recurrences and metastases caused by gastric cancer. Yet, the treatment process remains unknown. We aim to evaluate the potential pharmacological mechanism of JPHTF in interfering with resistance to trastuzumab in HER2‐positive gastric cancer (GC).

**Methods:**

In this study, network pharmacology and molecular docking techniques were used to forecast the potential active ingredients, pathways, and targets of JPHTF in overcoming trastuzumab resistance in HER2‐positive GC. Then, in vitro models of NCI‐N87/TR was developed, and JPHTF‐containing serum was utilized for intervention to confirm these crucial targets.

**Results:**

Network pharmacology showed that 92 potential active compounds and 420 therapeutic targets of JPHTF. SRC, EGFR, TP53, and AKT1 were identified as the main targets associated with the PI3K/Akt, MAPK, and Ras pathways, playing crucial roles in angiogenesis, cell apoptosis, cell proliferation, and resistance to chemotherapy in the GC microenvironment. Molecular docking analysis showed that quercetin, formononetin, and luteolin, which are the main active ingredients, exhibit high binding affinity to the central targets PI3K, AKT, and mTOR. In vitro experiment, the JPHTF‐containing serum has a significant alleviating effect on reversing trastuzumab resistance and cell apoptotic and proliferation of NCI‐N87/TR. Further molecular biological experiments showed that JPHTF could regulate the expression of PI3K/AKT/mTOR pathway.

**Conclusion:**

JPHTF has the ability to overcome trastuzumab resistance in NCI‐N87 cells through the regulation of the PI3K/AKT/mTOR pathway.

## Graphical abstract

Research methods of network pharmacology and molecular docking binding. Experimental validation was combined with network pharmacological analysis. In vitro study of NCI‐N87/TR cells in rat serum containing JPHTF. JPHTF reverses trastuzumab resistance in HER2‐positive GC through PI3K/AKT/mTOR pathway.

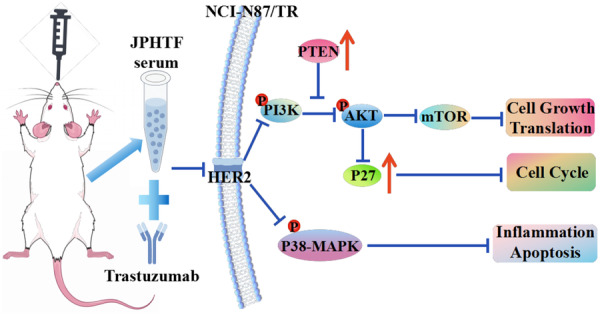

**Abbreviations**
AKT also known as PKB: protein kinase B
BC: betweenness centrality
BP: biological process
CC: closeness centrality
CHM: the Chinese herbal medicine
CNKI: China national knowledge infrastructure
DC: degree centrality
DL: drug likeness
EGFR: human epidermal growth factor receptor
GC: gastric cancer
GO: Gene Ontology
HER2: human epidermal growth factor receptor 2
JPHTF: Jian Pi Hua Tan Fang
KEGG: Kyoto Encyclopedia of Genes and Genomes
MF: molecular function
OB: oral bioavailability
OMIM: the Online Mendelian Inheritance in Man
PI3K: phosphatidylinositol‐3‐kinase
PPI: protein‐protein interaction
RCSB PDB: RCSB Protein Data Bank
TCM: traditional Chinese medicine
TCMID: traditional Chinese medicines integrated database
TCMSP: traditional Chinese medicine systems pharmacology database
UniProt: the Universal Protein Resource

## 1 Introduction

World Health Organization data shows that gastric cancer (GC) is the fifth most common malignant tumor and the fourth leading cause of cancer‐related deaths [1, 2]. Human epidermal growth factor receptor 2 (HER2) is a key component of the human epidermal growth factor receptor (EGFR) family [3]. HER2 is detected in about 10%–20% of cases of GC [4]. Ongoing research in tumor molecular targeted therapy has uncovered HER2 as a crucial factor contributing to poor prognosis in GC, particularly in the context of HER2‐positive advanced GC. Consequently, inhibiting the HER2 pathway has emerged as a fundamental strategy for front‐line treatment of HER2‐positive advanced GC. Blocking the HER2 pathway is now a fundamental approach for initial therapy of HER2‐positive advanced GC [5, 6]. As a first‐line drug targeting HER2 expression, trastuzumab has achieved good therapeutic effects [7]. However, its resistance limits its clinical application. As confirmed by the ToGA trial [8], trastuzumab combined with chemotherapy improves survival rates among patients with late‐stage GC overexpressing HER2. Drug resistance has developed within a year of treatment, with its resistance mechanism still not fully understood. It is imperative to address the issue of drug resistance promptly.

Existing studies have demonstrated that HER2 overexpression instigates alterations in signaling pathways, thereby instigating drug resistance [9, 10, 11]. Phosphatidylinositol‐3‐kinase (PI3K) is crucial in initiating the cascade reaction of the PI3K pathway and the continuous activation of protein kinase B (PKB, or AKT) in the progression of tumors. AKT is involved in many biological processes, including apoptosis, cell cycle regulation, and protein synthesis. AKT activation further stimulates the mammalian target of rapamycin (mTOR), which is an important regulator of intracellular energy metabolism and cell growth. Abnormal activation of the PI3K/AKT/mTOR signaling pathway can lead to unrestricted cell growth, blocked apoptosis, increased angiogenesis, and chemotherapy resistance, which are key factors in tumor occurrence and development [12, 13]. Prior research has indicated that the HER2‐mediated abnormal activation of the PI3K/AKT/mTOR pathway is essential in the development of resistance to trastuzumab in GC [14, 15]. Consequently, it has emerged as a prominent research area for intervening in HER2‐positive GC trastuzumab resistance. It is therefore of great clinical and social importance to seek effective strategies for reducing the susceptibility and intervening in the development of HER2‐positive GC.

Traditional Chinese medicine (TCM) possesses distinct characteristics, including the ability to target multiple entities and exhibit minimal side effects. The Jian Pi Hua Tan Fang (JPHTF) is a formula developed by Professor Youqi Xu [16]. Clinical studies have previously confirmed that combining JPHTF with chemotherapy significantly enhances patients' quality of life and exhibits favorable effects against GC recurrence and metastasis [17, 18]. Astragalus membranaceus and *Agrimonia eupatoria* in the prescription are used as sovereign drug to strengthen the spleen and replenish qi, while Coicis SDen and Polyporus Umbellatus are used as minister drug to relieve dampness and phlegm; Figwort Root, Fructus Ligustri Lucidi, and Caulis spatholobi support blood flow and nourish, whereas Hedyotis Diffusae Herba and Sophorae Flavescentis Radix aid in detoxification, all of which are assistant drug; accompanied by the passage of Radix Clematidis through the 12 meridians, and softening and dispersing knots, it plays a role in strengthening the spleen, resolving phlegm, and detoxifying cancer. However, the complex formula and diverse ingredients of JPHTF make it difficult to study its mechanism and effects. In vitro experiments and molecular docking were conducted to validate the treatment pathways and targets predicted by network pharmacology based on the construction of a component target gene network and topological analyses. By doing so, it is possible to study the interaction between JPHTF and HER2‐positive GC.

**Figure 1:**
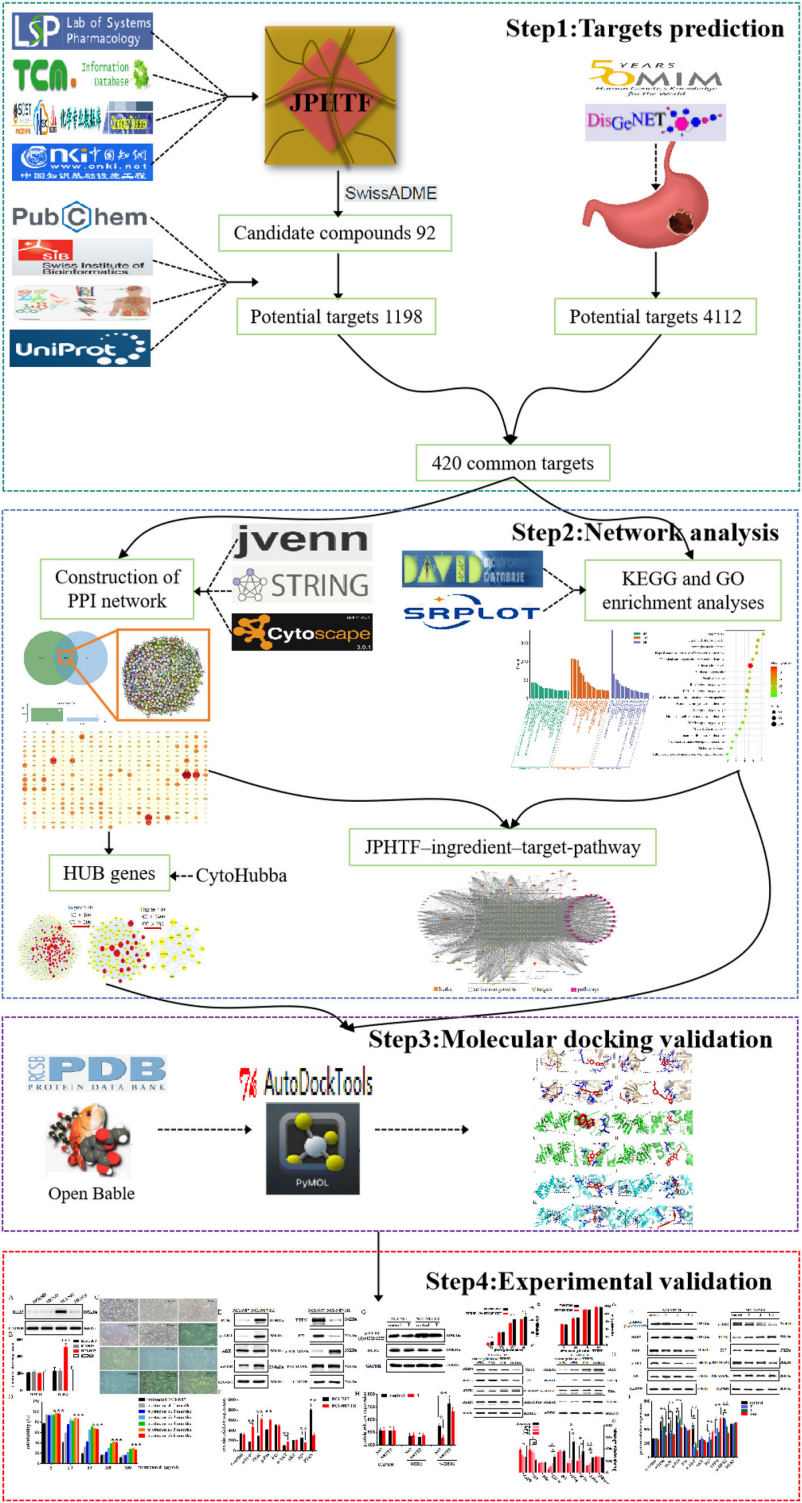
The workflow of the study.

We explored the potential mechanisms that may contribute to the resistance to trastuzumab of HER2‐positive GC in this study by applying network pharmacology techniques, including identifying active components and targets in JPHTF, searching HER2‐positive GC action sites, constructing PPI networks, KEGG and GO analysis. Next, the active components are docked with the anticipated targets using molecular docking. Finally, a HER2‐positive GC NCI‐N87 cell model with resistance to trastuzumab was constructed, and JPHTF‐containing serum was prepared. The improvement effect of JPHTF on the NCI‐N87/TR and its potential mechanism were verified through predicted targets. Figure 1 shows the detailed strategy for this study.

## 2 Materials and Methods

### 2.1 Bioactive Ingredients in JPHTF

Traditional Chinese medicine systems pharmacology database (TCMSP, https://tcmsp-e.com/index.php) [19] and traditional Chinese medicines integrated database (TCMID, https://bidd.group/TCMID/) [20] were used to source the ingredients for JPHTF. In the TCMSP database, bioactive ingredient screening was performed using the oral bioavailability (OB) ≥ 30% and the drug likeness (DL) ≥ 0.18 [21]. For those components obtained from TCMID, the Swiss ADME web service (http://www.swissadme.ch/) [22] conducted screening using computational methods to analyze the physicochemical properties and predict the pharmacokinetics, drug‐likeness, and suitability for medicinal chemistry of small compounds [23].

### 2.2 Bioactive Ingredient‐Related Targets

PubChem compound database (https://pubchem.ncbi.nlm.nih.gov/) [24] was used to obtain the Canonical SMILES corresponding to the screened bioactive ingredients. Next, a set of databases, including the Swiss Target Prediction database (http://www.swisstargetprediction.ch/) [25] and the TargetNet database (http://targetnet.scbdd.com/calcnet/index/) [26] are used to predict active compounds based on the biological parameter “*Homo sapiens.*” As a final step, UniProt IDs were searched through the Universal Protein Resource (UniProt) database (https://www.uniprot.org/) [27] for batch standardization.

### 2.3 Therapeutic Targets for HER2‐positive GC

By searching the DisGeNET database (https://www.disgenet.org/) [28] and the Online Mendelian Inheritance in Man(OMIM, https://omim.org/) [29] for keywords “HER2‐positive gastric cancer” and “*Homo sapiens*,” disease‐related targets were obtained.

### 2.4 “Herb‐Ingredient‐Target‐Pathway” Network Construction

An online Venn diagram mapping platform (https://bioinfogp.cnb.csic.es/tools/venny/) [30] was used to map the identified targets of JPHTF in the treatment of HER2‐positive GC. The region of intersection between the two circles of the Venn diagram indicates the potential targets of JPHTF. The “herb–ingredient–target‐pathway” network was created using Cytoscape v3.9.1 [31].

### 2.5 Protein–Protein Interaction Network

String database (STRING, https://cn.string-db.org/) [32] was used to conduct the protein‐protein interactions (PPI) network for “*Homo sapiens*” with a minimum interaction score of 0.700. After downloading the potential therapeutic gene protein interaction network data into Cytoscape and using the function of CytoHubba plug‐in, the data were sorted by degree centrality (DC), betweenness centrality (BC) and closeness centrality (CC) and then the key genes were selected [33].

### 2.6 GO and KEGG Enrichment Analysis

With the aid of the DAVID database (https://david.ncifcrf.gov/) [34], all potential therapeutic targets were enriched for terms from the Gene Ontology (GO) and pathway enrichment analyses from the Kyoto Encyclopedia of Genes and Genomes (KEGG). There are three categories of GO terms: biological process (BP), cellular component (CC), and molecular function (MF). We considered q‐values (enrichment score) under 0.05 as statistically significant and retained them. As a result of the count, the top 20 GO and KEGG enrichment results were visualized using SRplot‐Science and Research online plot (https://www.bioinformatics.com.cn/) [35] as bar graphs and bubble plots, respectively.

### 2.7 Molecular Docking

From the RCSB Protein Data Bank (RCSB PDB, http://www.pdb.org/) [36], we acquired the 3D protein structures of crucial goals. A crystalline arrangement of central elements was obtained from the PubChem database (https://pubchem.ncbi.nlm.nih.gov/) and converted to Mol2 format using Open Babel 2.4.1 [37]. Molecular docking was performed using AutoDockTools 1.5.6 after water was removed and the receptor protein was hydrotreated [38, 39]. Following this, the binding free energy (in kcal/mol) was determined as an indication of binding probability. According to general principles, binding energies ≤ −5.0 kcal/mol indicate good binding. A final visualization of the findings was made using PyMOL 2.6.0a0.

### 2.8 Cell Lines and Reagents

NCI‐N87 cell from Chinese Academy of Sciences Cell Bank (Shanghai, China) and MKN45 and MKN28 cell from Hongxin (Nanjing, China). The JPHTF is offered by the Nanjing Integrated Traditional Chinese and Western Medicine Hospital's Preparation Center (Nanjing, China); Trastuzumab, specification 440 mg per bottle, batch number N7479, from Roche; RMPI1640, MTS and BCA reagent kit from KeyGEN Bio TECH; Fetal bovine serum from Gibco; 0.25% trypsin, RIPA, goat anti rabbit and mouse IgG from Biosharp; 30% Acr Bis (29:1), TEMED, APS, PMSF, phosphorylase inhibitor and ECL chemiluminescence reagent kit from Beyotime; Pre stained protein Marker from Thermo Corporation; PVDF membrane (0.45 μm) from Merck Millipore; HER‐2 from abcam; AKT, p‐AKT, PI3K, mTOR, PTEN, P27 from Bioworld; GAPDH, P38‐MAPK, p‐P38‐MAPK from Cell Signaling.

### 2.9 Preparation of JPHTF‐Containing Serum

It was determined that the 40 SD rats would be split into two groups at random: the JPHTF group (*n* = 20) treated with JPHTF at a dosage of 210.4 g/kg/day and a volume of 20 mL/kg, equivalent to the clinical dosage for humans; and the blank group (*n* = 20) treated with an equivalent amount of saline. The drugs were administered via gastric gavage twice daily for three consecutive days. Fasting for 12 h before the last dose and collecting arterial blood from the abdominal aorta 2 h after the final dose. Following centrifugation for 15 min at 3000 r/min, a 56°C water bath was used to inactivate the serum fraction, and a 0.22 µm filter was used for filtration [40, 41, 42, 43]. At −80°C, the serum containing JPHTF was aliquoted and stored. The experimental animal research content involved in this project has been reviewed by the Experimental Animal Ethics Committee of Nanjing University of Chinese Medicine. It is in accordance with the Regulations on the Management of Experimental Animals issued by the State Science and Technology Commission, the Implementation Rules for the Management of Medical Experimental Animals issued by the Ministry of Health, the Measures for the Management of Experimental Animals of Jiangsu Province issued by the Government of Jiangsu Province, and the relevant regulations of the Experimental Animal Ethics Committee of Nanjing University of Chinese Medicine.

### 2.10 Cell Culture

The four GC cell lines (NCI‐N87, MGC‐803, MKN45, and MKN28) were cultivated in RPMI 1640 medium supplemented with 10% fetal bovine serum and 100 U/mL penicillin‐streptomycin. The cells were maintained at 37°C with 5% CO_2_ and were digested using a solution of 0.25% trypsin.

### 2.11 Establishment of Trastuzumab‐Resistant NCI‐N87/TR Cells

NCI‐N87 cells in 25 cm^2^ cell culture flask were treated with 44 μg/mL trastuzumab for 48 h, after 48 h of recovery, the trastuzumab was stimulated again and the process was repeated until the cells stabilized at this concentration of trastuzumab. By analogy, the induced concentrations are 44, 88, 250, 500, 1000, 1500, 2000, 3000 and 3600 μg/mL increases sequentially. Document the dosing date, capture images to monitor cell morphology alterations, and assess drug resistance at 4‐week intervals. After the concentration of 3600 μg/mL, until the cell state returns to normal, routine passage culture is carried out, and lastly, NCI‐N87 was injected with a concentration of 3000 μg/mL of trastuzumab. A stable growth in medium for 1 month will determine the success of the establishment of NCI‐N87/TR cells.

### 2.12 Cell Viability Assay

An equal number of NCI‐N87 or NCI‐N87/TR cells were plated in each well of a 96‐well plate at a density of 5 × 10^3^ cells per well. Following a 48‐h treatment period with the indicated concentrations of trastuzumab, JPHTF‐containing serum, or a combination thereof, a second incubation of 4 h was performed after adding 20 µL of MTS. A microplate reader was used to measure the absorbance at 490 nm, and GraphPad was used to calculate the half maximum inhibitory concentration (IC_50_). The inhibition factor (%) was calculated by taking [1 − (mean absorbance of the experimental group)/(mean absorbance of the control group)] × 100. Based on the IC_50_ value of resistant cells divided by the IC_50_ value of parental cells, the resistance index (RI) was calculated. The resistance reversal index was calculated using the formula: (IC_50_ in the trastuzumab group/IC_50_ in the combination group).

### 2.13 Western blot Analysis

In 6‐well plates, 2.5 × 10^5^ cells were seeded for western blot analysis. The following steps were performed: Following drug treatment, cells were harvested, subjected to RIPA lysis, and underwent ultrasonication for protein extraction. To determine the protein concentration, the BCA method was used, and the protein was denatured and stored at −20°C. Next, the proteins of varying molecular weights were separated using SDS‐PAGE gel electrophoresis, then transferred onto a PVDF membrane and left to incubate at 4°C with primary antibodies overnight. HRP‐labeled secondary antibodies were then added, followed by the application of ECL staining solution for color development.

### 2.14 Statistical Analysis

A minimum of three independent experiments were conducted, and the collected data was analyzed using GraphPad. Statistical data was expressed as mean and standard deviation. Statistical significance was defined as a difference with a *p*‐value less than 0.05, and substantial significance was defined as a difference with a *p*‐value less than 0.01.

## 3 Results

### 3.1 Screening of Common Targets Between JPHTF and Gastric Cancer

**Figure 2:**
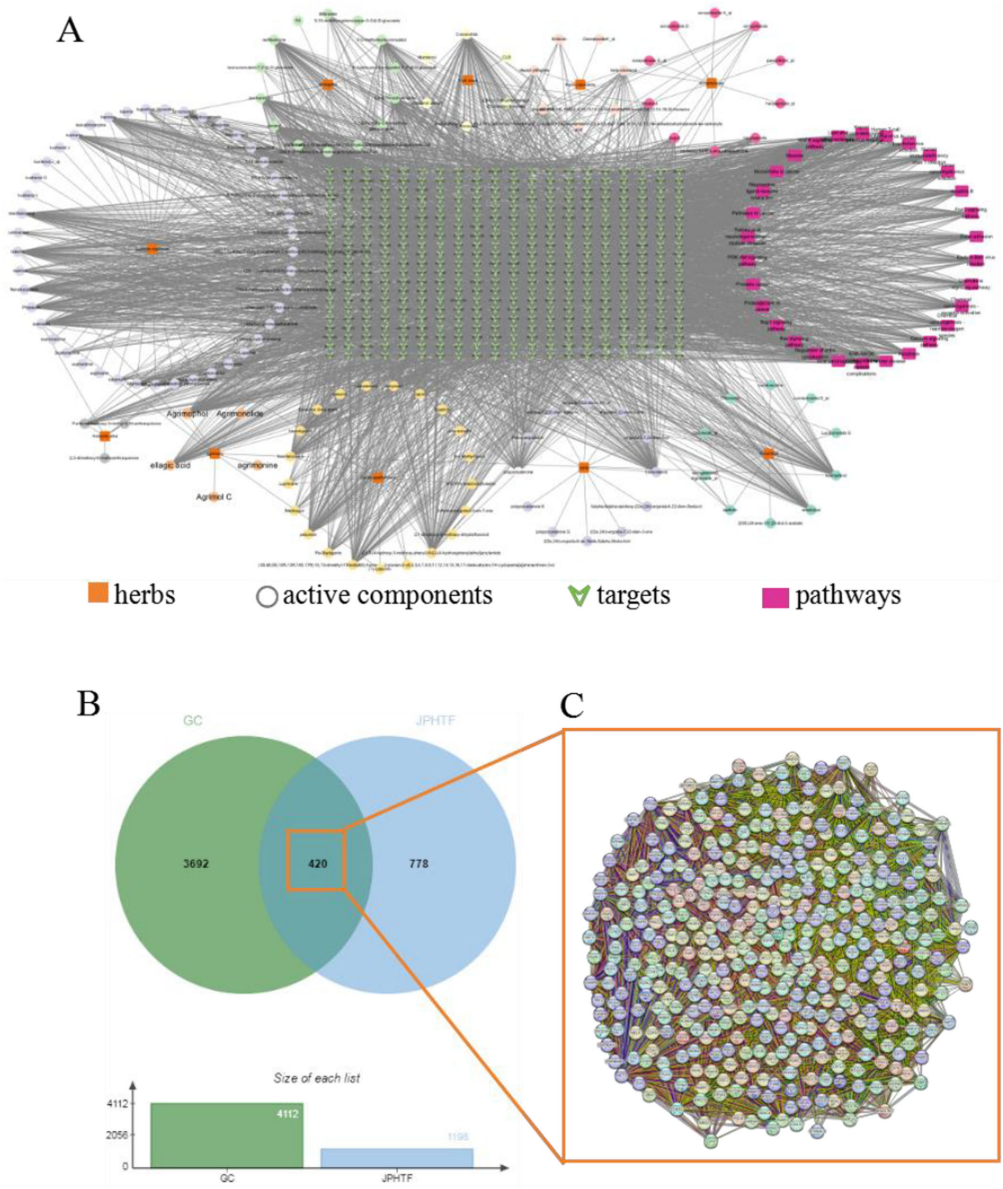
(A) The “herb–ingredient–target‐pathway” network of JPHTF. The tangerine square, circular, green arrow, and red rectangle corresponded to the herbs, active components, predicted targets, and top 20 pathways respectively. (B) Venn diagram showing the intersection of JPHTF‐related genes and GC‐related genes. The GC‐related targets are shown in the green circle, and the JPHTF‐related targets are shown in the blue circle. The intersection of the two circles indicates potential targets of JPHTF in GC treatment. (C) Protein–protein interaction (PPI) network of common targets were performed by STRING database, each edge represented the connection between targets.

JPHTF was composed of 10 TCMs (Supplementary Table 1). After eliminating duplicate values and those without targets, a total of 161 bioactive components were identified, with 92 being selected for further investigation in the subsequent study (Supporting Information S1: Table S2). In total, 4112 GC‐related targets and 1198 JPHTF‐related targets were obtained. Additionally, 420 shared targets were identified by comparing the targets of JPHTF and GC (Supporting Information S1: Table S3). Using the approach described, Cytoscape constructed the network “herb‐ingredient‐target‐pathway” as depicted in Figure 2A, consisting of 517 nodes and 4278 edges. Additionally, the primary five crucial components were acquired including quercetin (5280343), formononetin (5280378), luteolin (5280445), kaempferol (5280863), and hederagenin (73299). The shared targets network analysis was conducted using an online Venn diagram mapping platform (Figure 2B).

### 3.2 PPI Network Analysis and Hub Gene Identification

**Figure 3:**
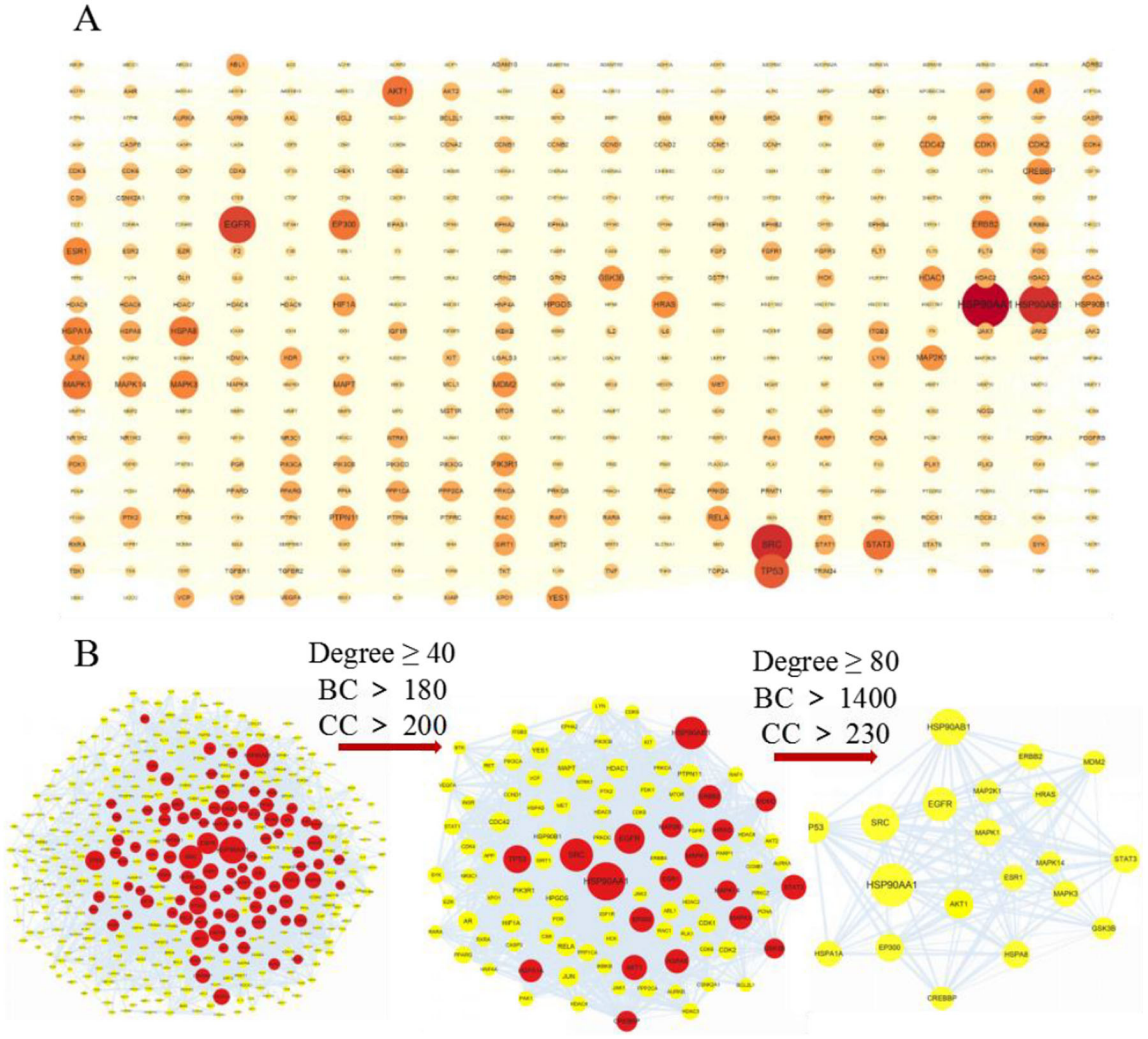
(A) The PPI network of the 420 cross‐targets were constructed using Cytoscape. Nodes represent targets, and lines represent interactions between two targets. PPI network: the darker the color, the larger the node, the larger its degree value and the greater its importance within the network. (B) PPI network hub gene screening process: The red nodes represent the targets that meet the screening conditions.

Based on the STRING database, we obtained a protein interaction network diagram showing potential targets of JPHTF intervention in HER2‐positive GC (Figure 2C), and a new PPI network comprising 410 nodes and 5673 edges was constructed by download and import Cytoscape software (Figure 3A). The mean node degree is 27.67, with an average central clustering coefficient of 0.344. The primary PPI network meeting the criteria (BC ≥ 80, CC > 230, DC > 1400) contained 20 nodes and 150 edges, with an average node degree of 108.25 (Figure 3B). HSP90AA1, SRC, EGFR, TP53, AKT1, STAT3, EP300, MAPK1, ERBB2, and ESR1 are identified as HUB genes in the core‐target PPI network due to their high degree values, potentially contributing significantly to the treatment process according to Table 1.

**Table 1:** DC, BC, CC, and stress information of HUB genes.

Gene Name	DC	BC	CC	Stress
HSP90AA1	184	10765.89653	292.91667	149,122
SRC	154	9656.28483	279	124,448
EGFR	138	9068.8004	270.33333	111,904
TP53	124	7191.4298	261.83333	92,518
HSP90AB1	152	5779.23612	275.75	96,678
STAT3	108	4157.57861	253.91667	60,258
ESR1	94	3756.2217	245.08333	54,010
ERBB2	97	3701.1586	246.08333	47,670
HSPA8	103	3609.60382	250.41667	47,270
AKT1	109	3451.9212	254	54,650
EP300	107	3293.96685	251.08333	52,488
MDM2	83	2886.80153	239.75	28,846
MAPK3	97	2847.13446	246.91667	51,170
HSPA1A	96	2780.74947	247	50,586
MAPK1	102	2408.26308	250.83333	60,630
MAP2K1	84	2292.24162	238.08333	41,946
HRAS	88	2245.0238	240.25	34,742
GSK3B	80	2127.62868	235.66667	40,192
MAPK14	81	1455.08487	238.41667	35,360
CREBBP	84	1408.87343	238.58333	27,924

### 3.3 GO Functional Enrichment and KEGG Pathway Analysis

After removing data with a *p*‐value of ≥ 0.05 that was not related to GC, a total of 990 BP, 120 CC, 229 MF, and 170 KEGG pathways were identified. A bar or bubble diagram displayed the most important 20 GO terms in BP, CC, and MF (Supporting Information S1: Table S4), as well as the top 20 significantly enriched KEGG pathways (Supporting Information S1: Table S5).

**Figure 4:**
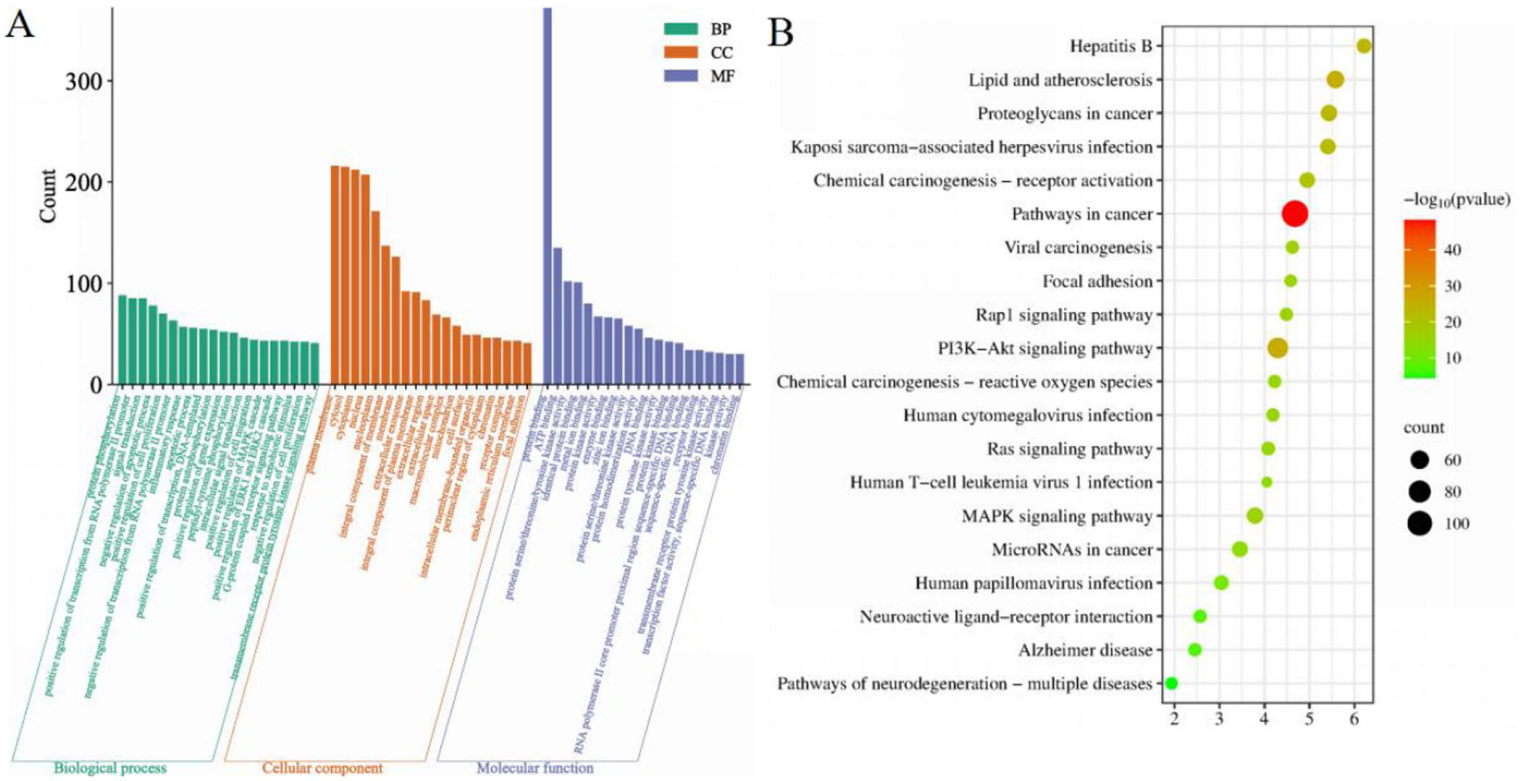
(A) Barplot performed the top 20 enriched terms of BP, CC, and MF. The horizontal axis represented the name of each term, and the vertical axis represented the counts of genes in the enriched term. (B) The bubble plots of the 20 most significant signaling pathways based on KEGG enrichment analysis. The bubble size represents the number of genes enriched in this pathway, the bubble color difference represents the level of gene enrichment in this pathway, and the circle size represents the number of targets contained in the pathway. The redder the color, the smaller the *p* value.

BP related to the management of JPHTF in HER2‐positive GC primarily correlated with protein phosphorylation response, signal transduction response, inhibition of apoptosis, and promotion of cell growth. The CC ontology primarily focused on plasma membrane, cytosol, cytoplasm, nucleus, and nucleoplasm for the targets. Within the MF ontology, the focuses of JPHTF on HER2‐positive GC primarily involved interactions with proteins, ATP, and protein kinases, including serine/threonine/tyrosine kinase activity and identical protein binding (Figure 4A). Analysis of KEGG enrichment revealed that JPHTF had a significant impact on pathways such as PI3K‐Akt signaling, proteoglycans, and MAPK signaling (Figure 4B).

### 3.4 Interaction of Central Targets With Bioactive Compounds Through Molecular Docking

**Figure 5:**
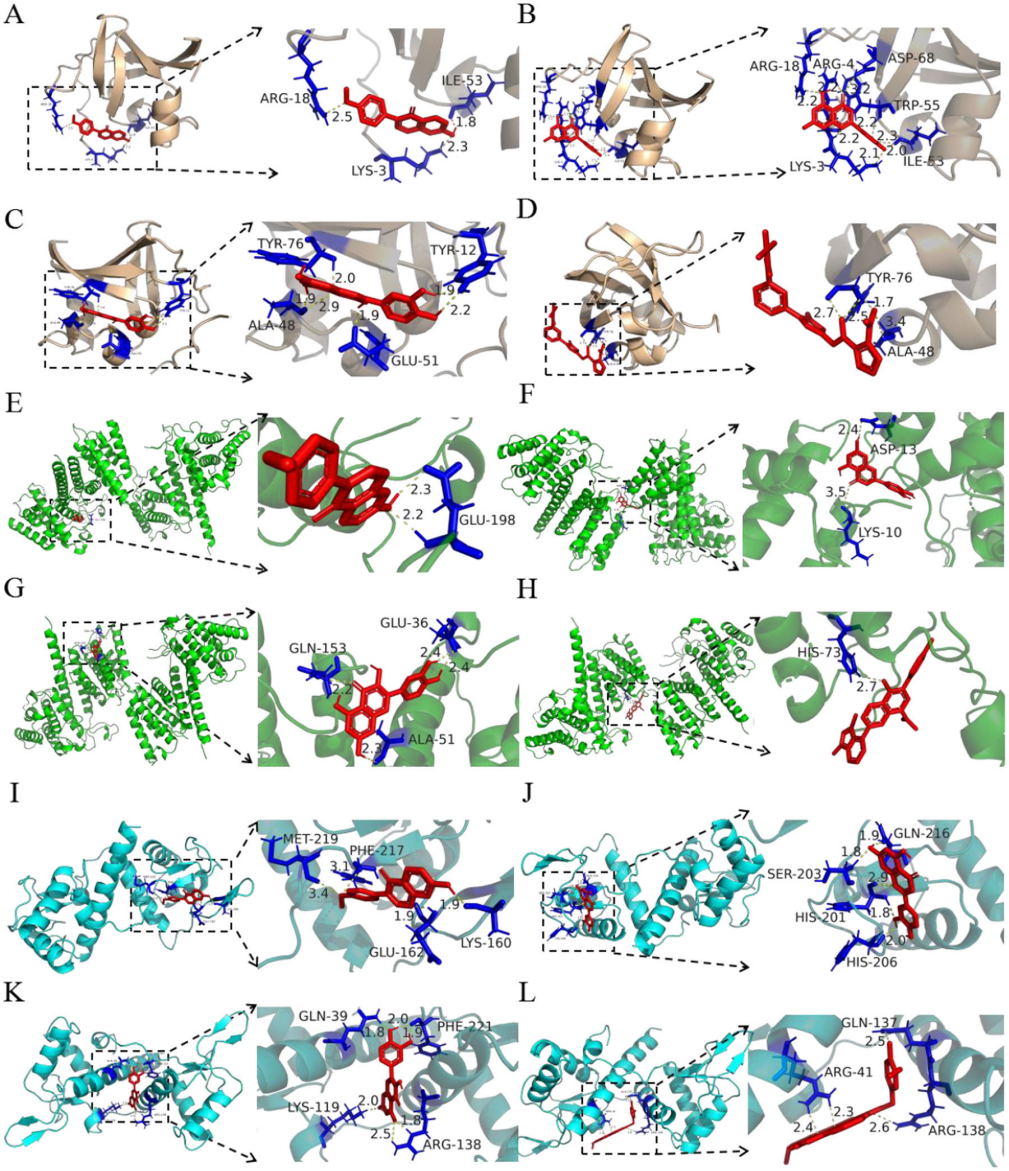
Molecular docking of hub targets and active components of JPHTF. (A) PI3K‐formononetin (binding site: ARG‐18, ILE‐53, LYS‐3); (B) PI3K‐luteolin (binding site: ARG‐18, ARG‐4, ILE‐53, LYS‐3, TRP‐55, ASP‐68); (C) PI3K‐quercetin (binding site: TYR‐76, TYR‐12, ALA‐48, GLU‐51); (D) PI3K‐alpelisib (PI3K inhibitor) (binding site: TYR‐76, ALA‐48); (E) AKT‐formononetin (binding site: GLU‐198); (F) AKT‐luteolin (binding site: LYS‐10, ASP‐13); (G) AKT‐quercetin (binding site: GLN‐153, GLU‐36, ALA‐51); (H) AKT‐ipatasertib (AKT inhibitor) (binding site: HIS‐73); (I) mTOR‐formononetin (binding site: MET‐219, PHE‐217, GLU‐162, LYS‐160); (J) mTOR‐luteolin (binding site: SER‐203, GLN‐216, HIS‐201, HIS‐206); (K) mTOR‐quercetin (binding site: GLN‐39, LYS‐119, PHE‐221, ARG‐138); (L) mTOR‐rapamycin (mTOR inhibitor) (binding site: GLN‐137, ARG‐138, ARG‐41).

According to the screened core targets and the results of KEGG analysis, JPHTF most likely affected HER2‐positive GC by regulating the PI3K/AKT signaling pathway. Therefore, we used the active components of JPHTF (quercetin, formononetin, and luteolin) and positive controls (alpelisib, ipatasertib, and rapamycin, which are inhibitors of PI3K, AKT, and mTOR, respectively) as the ligands. The receptors used were PI3K (PDB: 3I5R), AKT (PDB: 5FIA), and mTOR (PDB: 7DKL). Molecular docking was carried out using AutoDockTools, and visualization using PyMOL, as shown in Table 2. The results of molecular docking indicated that the calculated binding energies for the primary active ingredients and crucial therapeutic targets were below −5 kcal/mol, suggesting potential specific binding. When quercetin, formononetin, and luteolin bind to the PI3K, AKT, and mTOR proteins, respectively, they interact with amino acid residues in the binding domains of these proteins via hydrogen bonding. Some key amino acids, such as ARG and TYR in PI3K, and ARG and GLN in AKT and mTOR, play a crucial role in binding to the active components of JPHTF. In Figure 5, the visual representations and specific information regarding the ideal connection of important targets to the active elements of JPHTF are displayed. The findings suggested that key components of JPHTF could effectively interact with the receptor proteins of HER2‐positive GC, potentially influencing the PI3K‐Akt signaling pathway.

**Table 2:** Molecular docking model information and docking results.

Drug	Targets	PDB ID	Original ligand	Binding energy (kcal/mol)
Alpelisib	PI3K	3I5R	—	−6.77
Ipatasertib	AKT	5FIA	2‐(N‐MORPHOLINO)‐ETHANESULFONIC ACID (MES), (4S)‐2‐METHYL‐2,4‐PENTANEDIOL (MPD)	−4.95
Rapamycin	mTOR	7DKL	—	−8.29
Quercetin	PI3K	3I5R	—	−6.97
Quercetin	AKT	5FIA	2‐(N‐MORPHOLINO)‐ETHANESULFONIC ACID (MES), (4S)‐2‐METHYL‐2,4‐PENTANEDIOL (MPD)	−5.32
Quercetin	mTOR	7DKL	—	−7.48
Formononetin	PI3K	3I5R	—	−7.03
Formononetin	AKT	5FIA	2‐(N‐MORPHOLINO)‐ETHANESULFONIC ACID (MES), (4S)‐2‐METHYL‐2,4‐PENTANEDIOL (MPD)	−6.29
Formononetin	mTOR	7DKL	—	−6.16
Luteolin	PI3K	3I5R	—	−7.1
Luteolin	AKT	5FIA	2‐(N‐MORPHOLINO)‐ETHANESULFONIC ACID (MES), (4S)‐2‐METHYL‐2,4‐PENTANEDIOL (MPD)	−5.49
Luteolin	mTOR	7DKL	—	−7.32

### 3.5 Investigating HER2‐Positive Gastric Cancer Cell Lines to Induce Resistance to Trastuzumab and Study the Underlying Resistance Mechanisms

**Figure 6:**
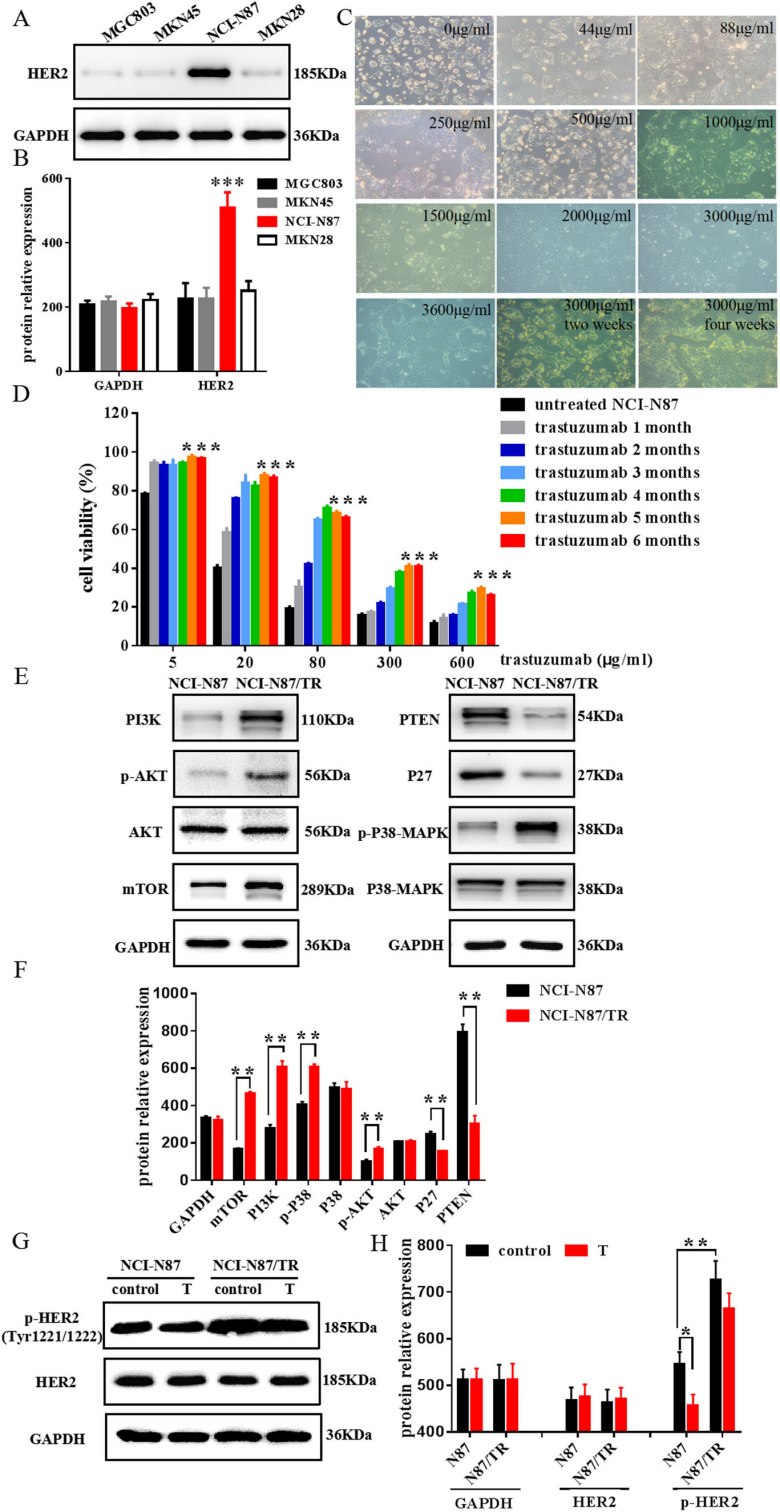
NCI‐N87/TR construction, preliminary investigation of trastuzumab resistance. (A) Examining HER2 using Western blot in MGC803, MKN145, NCI‐N87, and MKN28 cell lines; (B) HER2 expression levels were measured relative to GAPDH through normalization; (C) Trastuzumab induces morphological changes in NCI‐N87 cells; (D) MTS assay was used to detect the viability of NCI‐N87 cells treated with trastuzumab.; (E) and (F) NCI‐N87 and NCI‐N87/TR cells were subjected to Western blot analysis for PI3K, p‐AKT, AKT, mTOR, PTEN, P27, P38‐MAPK, and p‐P38‐MAPK; (G) and (H) NCI‐N87 and NCI‐N87/TR cells, both treated and untreated with trastuzumab, were subjected to Western blot analysis for HER2 and p‐HER2 (Tyr1221/1222). Results are shown as the average ± standard deviation (*n* = 3), with statistical significance indicated by *p < 0.01, **p < 0.001 when compared to the control group.

Figure 6A,B demonstrate that NCI‐N87 has the highest level of HER2 protein expression. Based on that, we selected NCI‐N87 cells with high HER2 expression levels as the research object for the next step of drug resistance cell induction experiments.

The creation of a trastuzumab‐resistant cell line called NCI‐N87/TR for human HER2‐positive gastric cancer was achieved successfully (Figure 6C). The MTS results showed that with the enhancement of NCI‐N87 cell resistance, the cell survival rate showed a significant upward trend (Figure 6C), and the IC_50_ of NCI‐N87 or NCI‐N87/TR to trastuzumab were 14.35 or 190.3 μg/ml, respectively. Calculate the drug resistance index at different drug resistance stages, and the ultimately induced drug‐resistant strain NCI‐N87/TR has a resistance index of 13.26 (Supporting Information S1: Table S6). Western blot analysis revealed a notable increase in the levels of p‐AKT, p‐P38‐MAPK, mTOR, and PI3K proteins in NCI‐N87/TR cells compared to untreated NCI‐N87 cells, along with a significant decrease in the levels of P27 and PTEN proteins (Figure 6E,F). Meanwhile, we detected the expression levels of HER2 and phosphorylated HER2 (Tyr1221/1222) in trastuzumab‐treated and untreated NCI‐N87 and NCI‐N87/TR cells (Figure 6G,H). It was found that trastuzumab treatment reduced the expression of p‐HER2 (Tyr1221/1222) protein in NCI‐N87 cells, while NCI‐N87/TR cells increased the expression of p‐HER2 (Tyr1221/1222) protein relative to NCI‐N87 cells.

### 3.6 JPHTF Could Reverse Resistance of NCI‐N87 Cells to Trastuzumab by Regulating PI3K/AKT/mTOR Signaling Pathway

**Figure 7:**
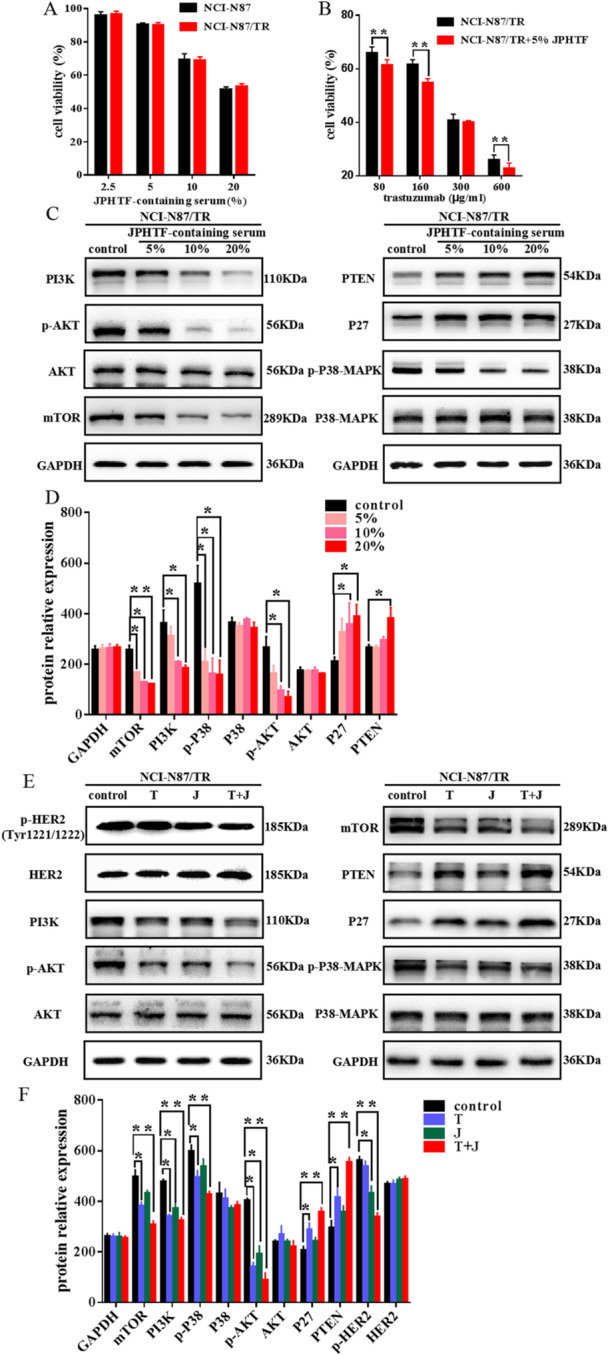
(A) The cell viability of NCI‐N87 and NCI‐N87/TR cells at diferent concentrations of JPHTF containing serum. (B) The cell viability of NCI‐N87/TR cells containing 5% JPHTF serum at different concentrations of trastuzumab. (C) NCI‐N87/TR celll western blot analysis of PI3K, p‐AKT, AKT, mTOR, PTEN, P27, P38‐MAPK and p‐P38‐MAPK at 48 h of culture with 5% blank serum and different JPHTF containing serum. (D) The relative protein levels of (C). (E) NCI‐N87/TR celll western blot analysis above proteins with 190 μg/mL trastuzumab, 5% JPHTF‐containing serum or combination of both, respectively. (F) The relative protein levels of (E). T: trastuzumab, J: JPHTF, T + J: trastuzumab + JPHTF. Data are presented as the mean ± SD (*n* = 6), *p < 0.05, **p < 0.01, ***p < 0.001 compared to the control group.

MTS analysis indicated that the JPHTF significantly suppressed the growth of NCI‐N87 and NCI‐N87/TR cells, with cell viability decreasing as drug concentration increased. The IC_50_ of the two cells were 19.91% and 21.88%, respectively, and no significant difference (Figure 7A). Subsequently, only NCI‐N87/TR cells were used for relevant mechanism research. It was found that when the serum concentration of JPHTF was 5%, the survival rate of NCI‐N87/TR cells could reach over 90%, so 5% is used as the combined concentration of JPHTF and trastuzumab. The IC_50_ of NCI‐N87/TR after combination therapy was 167.4 μg/mL and the trastuzumab alone was 190.3 μg/mL (Figure 7B). Subsequently, western blot analysis indicated a notable decrease in the protein levels of mTOR, p‐AKT, p‐P38‐MAPK, and PI3K, with no significant alterations observed in AKT and P38‐MAPK proteins. Conversely, the levels of P27 and PTEN proteins exhibited an increase in response to escalating drug concentration (Figure 7C,D).

Additionally, we confirmed the impact of the JPHTF and trastuzumab combination on protein levels in NCI‐N87/TR cell lines. Compared to the control group, administration of the combination led to a significant reduction in the expression of p‐HER2 (Tyr1221/1222), mTOR, PI3K, p‐P38‐MAPK, and p‐AKT, while displaying an even more pronounced decrease. In contrast, P27 and PTEN exhibited a significant increase (Figure 7E,F). These results suggest that the combined treatment of JPHTF and trastuzumab effectively promotes cell death by activating the PI3K/AKT/mTOR signaling pathway, consequently reversing trastuzumab resistance.

## 4 Discussion

As yet, the overexpression or amplification of HER2 is the sole established predictive biomarker for targeted therapy in GC [44, 45]. While trastuzumab has demonstrated improved overall survival (OS) and progression‐free survival (PFS) in HER2‐positive advanced GC [46], resistance to trastuzumab remains a significant concern for the majority of patients [10, 47, 48]. Although there have been recent advancements in reversing trastuzumab resistance in HER2‐positive GC, the complexity of its etiology and pathogenic mechanisms limits the availability of practical interventions. TCM has demonstrated effective treatment for trastuzumab resistance in HER2‐positive GC due to its unique properties involving multiple components, targets, and pathways. In our study, we discovered, for the first time, the reversal effects of JPHTF on trastuzumab resistance in HER2‐positive GC NCI‐N87/TR cell lines.

Furthermore, previous studies have revealed the potential of effective components found in JPHTF to reverse multidrug resistance. For instance, quercetin improves multidrug resistance in GC by inhibiting the expression and activity of permeable glycoprotein (P‐gp) of ABC transporters in the PI3K/Akt/P‐gp cascade [49]. As a potential tyrosine kinase inhibitor (TKI), luteolin inhibits RTKs‐HER2 receptor proteins to treat HER2‐positive GC [50]. Catechin, on the other hand, attenuates the overactivation of the HER2‐SHCBP1‐PLK1 axis and increases the sensitivity of HER2‐positive GC to trastuzumab [51].

Studies have also indicated that PI3K activation serves as an unfavorable prognostic factor for HER2‐positive GC [52, 53], and the PI3K/Akt/mTOR pathway acts as a predictive factor for HER2‐positive advanced GC treatment with trastuzumab [14]. To comprehensively investigate the PI3K/Akt/mTOR activation pathway, considering the prevalence of PTEN loss of expression within this pathway, we conducted Western blot analysis to assess PTEN expression. This was conducted due to the significance of posttranscriptional regulation in PTEN functionality [54, 55, 56]. Our analysis revealed a significant reduction in the protein expression level of the PTEN gene in NCI‐N87/TR cells compared to NCI‐N87 cells, alongside activated PI3K/Akt/mTOR signaling pathway. This suggests that downregulation of PTEN gene expression and excessive activation of the PI3K/Akt/mTOR signaling pathway may contribute to HER2‐positive GC resistance to trastuzumab.

**Figure 8:**
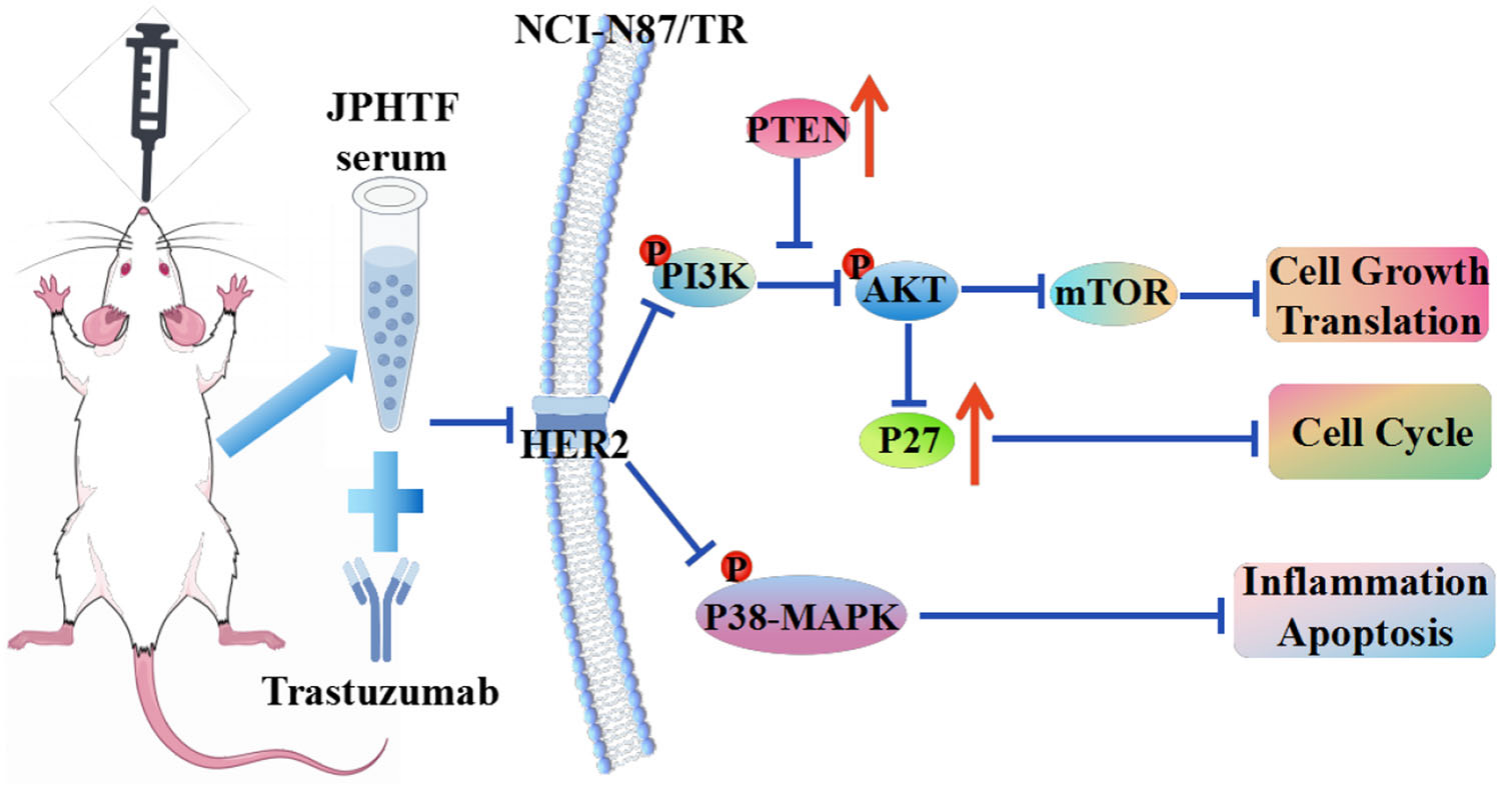
Schematic representation of Jian Pi Hua Tan Fang reverses trastuzumab resistance of HER2‐positive gastric cancer through PI3K/AKT/mTOR pathway. In the in vitro experiments, JPHTF serum noticeably enhances trastuzumab sensitivity, upregulates PTEN and P27 expression, effectively inhibits the activation of the PI3K/Akt/mTOR pathway, and subsequently reverses NCI‐N87 cell resistance to trastuzumab.

To further explore the mechanism through which JPHTF reverses trastuzumab resistance in NCI‐N87 cell, we evaluated the effects of varying concentrations of JPHTF and the combination of JPHTF and trastuzumab on NCI‐N87/TR cells. Our results demonstrated a gradient decrease in the protein phosphorylation level of the PI3K/Akt/mTOR signaling pathway with increasing concentrations of JPHTF. Additionally, P27 and PTEN exhibited increasing expression levels. Notably, the combination of JPHTF and trastuzumab generated even more significant changes. These findings indicate that JPHTF noticeably enhances trastuzumab sensitivity, upregulates PTEN expression, effectively inhibits the activation of the PI3K/Akt/mTOR pathway, and subsequently reverses NCI‐N87 cell resistance to trastuzumab (Figure 8).

The clinical application of TCM can benefit from the synergistic effects of its constituents, thereby reducing adverse reactions and enhancing the clinical efficacy compared to single drug treatments. However, there were still limitations in this study. It is essential to conduct further in vivo experimental studies to unravel the molecular mechanisms underlying the clinical therapeutic effects of JPHTF on GC, as this study is limited to in vitro experiments. In addition, the potential compounds involved in the JPHTF reversal of trastuzumab resistance in HER2‐positive gastric cancer need to be identified and validated in vivo or in vitro. Therefore, in our future research direction, NCI‐N87/TR cells will be injected into nude mice to directly evaluate the in vivo efficacy of JPHTF. At the same time, the UPLC‐MS/MS assay was used to screen the active components of JPHTF in the serum, and the key potential compounds involved in reversing trastuzumab resistance of JPHTF in HER2‐positive gastric cancer were identified and validated in vivo and vitro. As a team of researchers, we strive to establish a systematic drug analysis method that incorporates network pharmacology, multiple target molecular docking, and protein synthesis, as well as experimental verification in vitro, to maximize the effectiveness of TCM therapy, and the purpose of this is to provide a possible theoretical and experimental basis for standardizing and internationalizing TCM.

## 5 Conclusion

Using network pharmacology and molecular docking, we predicted that JPHTF regulates Akt, PI3K, and mTOR through the PI3K/Akt/mTOR pathway, thus reversing trastuzumab resistance in HER2‐positive gastric cancer. As a result of further experiments, it was confirmed that NCI‐N87/TR cell displayed inhibition of the PTEN gene as well as abnormal activation of the PI3K/Akt/mTOR pathway. Our findings identified that JPHTF was a potential strategy for preventing and effectively reversing the phenomenon of multidrug resistance in chemotherapy. It may serve as a potential chemotherapy sensitizer for the treatment of HER2‐positive GC that deserves further investigation.

## Author Contributions

**Jia Hu:** formal analysis, methodology; writing – original draft. **Wenjing Bu:** methodology, software, visualization. **Yongfang Ding:** data curation, validation, writing – original draft. **Xin Li:** formal analysis, validation. **Bo Zhang:** methodology, visualization. **Bo Shen:** supervision, validation. **Cong Wu:** Data curation, supervision. **Youqi Xu:** funding acquisition, project administration, resources. **Xiaoyang Zhang:** conceptualization, methodology, writing – original draft, writing – review and editing.

## Ethics Statement

The authors have nothing to report.

## Consent

The authors have nothing to report.

## Conflicts of Interest

The authors declare no conflicts of interest.

## Supporting information

Supporting information.

## Data Availability

The authors declare that they have no known competing financial interests or personal relationships that could have appeared to influence the work reported in this paper.
