# Supplementary material for: Jian Pi Hua Tan Fang Reverses Trastuzumab Resistance of HER2‐Positive Gastric Cancer Through PI3K/AKT/mTOR Pathway: Integrating Network Pharmacology, Molecular Docking and Experimental Validation
Source: Immun Inflamm Dis. 2025 Feb 7;13(2):e70154. doi: 10.1002/iid3.70154 (PMC11803458; doi:10.1002/iid3.70154)
Supplement: Supplementary file 1 — Supporting information. [file IID3-13-e70154-s001.docx]

**Supporting Information**

**Jian Pi Hua Tan Fang reverses trastuzumab resistance of HER2-positive gastric cancer through PI3K/AKT/mTOR pathway: integrating network pharmacology, molecular docking and experimental validation**

**Supplementary Table 1 Ingredients of JPHTF**

| **Ingredients** | **Weught(g)** |
| --- | --- |
| *Hedysarum Multijugum Maxim* (“Huangqi” in Chinese, HQ) | 30 |
| *Agrimonia Eupatoria* (“Xianhecao” in Chinese, XHC) | 30 |
| *Coicis Semen* (“Yiyiren” in Chinese, YYR) | 30 |
| *Polyporus Umbellatus* (“Zhuling” in Chinese, ZL) | 15 |
| *Figwort Root* (“Xuanshen” in Chinese, XS) | 15 |
| *Fructus Ligustri Lucidi* (“Nvzhenzi” in Chinese, NZZ) | 20 |
| *Spatholobus Suberectus Dunn* (“Jixueteng” in Chinese, JXT) | 30 |
| *Hedyotis Diffusae Herba* (“Baihuasheshecao” in Chinese, BHSSC) | 30 |
| *Sophorae Flavescentis Radix* (“Kushen” in Chinese, KS) | 15 |
| *Radix Clematidis* (“Weilingxian” in Chinese, WLX) | 30 |

**Supplementary Table 2 Bioactive ingredients of JPHTF**

| **Number** | **Pubchem Cid** | [**Molecule Name**](https://tcmsp-e.com/tcmspsearch.php?qr=Hedysarum%20Multijugum%20Maxim.&qsr=herb_en_name&token=20be72cbdaf6d2b31e618b69e3e16968) | [**MW**](https://tcmsp-e.com/tcmspsearch.php?qr=Hedysarum%20Multijugum%20Maxim.&qsr=herb_en_name&token=20be72cbdaf6d2b31e618b69e3e16968) | [**OB (%)**](https://tcmsp-e.com/tcmspsearch.php?qr=Hedysarum%20Multijugum%20Maxim.&qsr=herb_en_name&token=20be72cbdaf6d2b31e618b69e3e16968) | [**DL**](https://tcmsp-e.com/tcmspsearch.php?qr=Hedysarum%20Multijugum%20Maxim.&qsr=herb_en_name&token=20be72cbdaf6d2b31e618b69e3e16968) | **Source** |
| --- | --- | --- | --- | --- | --- | --- |
| 1 | [5280343](http://pubchem.ncbi.nlm.nih.gov/summary/summary.cgi?cid=5280343) | [quercetin](https://tcmsp-e.com/molecule.php?qn=98) | 302.25 | 46.43 | 0.28 | *Hedysarum Multijugum Maxim, Agrimonia Eupatoria, Fructus Ligustri Lucidi, Hedyotis Diffusae Herba, Sophorae Flavescentis Radix, Radix Clematidis* |
| 2 | [5280863](http://pubchem.ncbi.nlm.nih.gov/summary/summary.cgi?cid=5280863) | [kaempferol](https://tcmsp-e.com/molecule.php?qn=422) | 286.25 | 41.88 | 0.24 | *Hedysarum Multijugum Maxim, Agrimonia Eupatoria, Fructus Ligustri Lucidi* |
| 3 | [5280378](http://pubchem.ncbi.nlm.nih.gov/summary/summary.cgi?cid=5280378) | [formononetin](https://tcmsp-e.com/molecule.php?qn=392) | 268.28 | 69.67 | 0.21 | *Hedysarum Multijugum Maxim, Spatholobus Suberectus Dunn, Sophorae Flavescentis Radix* |
| 4 | [73299](https://pubchem.ncbi.nlm.nih.gov/compound/73299) | [hederagenin](https://tcmsp-e.com/molecule.php?qn=296) | 472.7 | 36.91 | 0.75 | *Hedysarum Multijugum Maxim, Spatholobus Suberectus Dunn* |
| 5 | [5280448](http://pubchem.ncbi.nlm.nih.gov/summary/summary.cgi?cid=5280448) | [Calycosin](https://tcmsp-e.com/molecule.php?qn=417) | 284.28 | 47.75 | 0.24 | *Hedysarum Multijugum Maxim, Spatholobus Suberectus Dunn* |
| 6 | [15976101](http://pubchem.ncbi.nlm.nih.gov/summary/summary.cgi?cid=15976101) | [(3S,8S,9S,10R,13R,14S,17R)-10,13-dimethyl-17-[(2R,5S)-5-propan-2-yloctan-2-yl]-2,3,4,7,8,9,11,12,14,15,16,17-dodecahydro-1H-cyclopenta[a]phenanthren-3-ol](https://tcmsp-e.com/molecule.php?qn=33) | 428.82 | 36.23 | 0.78 | *Hedysarum Multijugum Maxim, Spatholobus Suberectus Dunn* |
| 7 | [64971](http://pubchem.ncbi.nlm.nih.gov/summary/summary.cgi?cid=64971) | [Mairin](https://tcmsp-e.com/molecule.php?qn=211) | 456.78 | 55.38 | 0.78 | *Hedysarum Multijugum Maxim* |
| 8 | [5318869](http://pubchem.ncbi.nlm.nih.gov/summary/summary.cgi?cid=5318869) | [Jaranol](https://tcmsp-e.com/molecule.php?qn=239) | 314.31 | 50.83 | 0.29 | *Hedysarum Multijugum Maxim* |
| 9 | [5281654](http://pubchem.ncbi.nlm.nih.gov/summary/summary.cgi?cid=5281654) | [isorhamnetin](https://tcmsp-e.com/molecule.php?qn=354) | 316.28 | 49.6 | 0.31 | *Hedysarum Multijugum Maxim* |
| 10 | [15689655](http://pubchem.ncbi.nlm.nih.gov/summary/summary.cgi?cid=15689655) | [3,9-di-O-methylnissolin](https://tcmsp-e.com/molecule.php?qn=371) | 314.36 | 53.74 | 0.48 | *Hedysarum Multijugum Maxim* |
| 11 | [15689652](http://pubchem.ncbi.nlm.nih.gov/summary/summary.cgi?cid=15689652) | [7-O-methylisomucronulatol](https://tcmsp-e.com/molecule.php?qn=378) | 316.38 | 74.69 | 0.3 | *Hedysarum Multijugum Maxim* |
| 12 | [14077830](http://pubchem.ncbi.nlm.nih.gov/summary/summary.cgi?cid=14077830) | [(6aR,11aR)-9,10-dimethoxy-6a,11a-dihydro-6H-benzofurano[3,2-c]chromen-3-ol](https://tcmsp-e.com/molecule.php?qn=380) | 300.33 | 64.26 | 0.42 | *Hedysarum Multijugum Maxim* |
| 13 | [108213](http://pubchem.ncbi.nlm.nih.gov/summary/summary.cgi?cid=108213) | [Bifendate](https://tcmsp-e.com/molecule.php?qn=387) | 418.38 | 31.1 | 0.67 | *Hedysarum Multijugum Maxim* |
| 14 | [160767](https://pubchem.ncbi.nlm.nih.gov/compound/160767) | [isoflavanone](https://tcmsp-e.com/molecule.php?qn=398) | 224.25 | 109.99 | 0.3 | *Hedysarum Multijugum Maxim* |
| 15 | [6037](http://pubchem.ncbi.nlm.nih.gov/summary/summary.cgi?cid=6037) | [FA](https://tcmsp-e.com/molecule.php?qn=433) | 441.45 | 68.96 | 0.71 | *Hedysarum Multijugum Maxim* |
| 16 | [10380176](http://pubchem.ncbi.nlm.nih.gov/summary/summary.cgi?cid=10380176) | [(3R)-3-(2-hydroxy-3,4-dimethoxyphenyl)chroman-7-ol](https://tcmsp-e.com/molecule.php?qn=438) | 302.35 | 67.67 | 0.26 | *Hedysarum Multijugum Maxim* |
| 17 | [15689653](http://pubchem.ncbi.nlm.nih.gov/summary/summary.cgi?cid=15689653) | [isomucronulatol-7,2'-di-O-glucosiole](https://tcmsp-e.com/molecule.php?qn=439) | 626.67 | 49.28 | 0.62 | *Hedysarum Multijugum Maxim* |
| 18 | [5316760](http://pubchem.ncbi.nlm.nih.gov/summary/summary.cgi?cid=5316760) | [1,7-Dihydroxy-3,9-dimethoxy pterocarpene](https://tcmsp-e.com/molecule.php?qn=442) | 314.31 | 39.05 | 0.48 | *Hedysarum Multijugum Maxim* |
| 19 | [5280445](http://pubchem.ncbi.nlm.nih.gov/summary/summary.cgi?cid=5280445) | [luteolin](https://tcmsp-e.com/molecule.php?qn=6) | 286.25 | 36.16 | 0.25 | *Agrimonia Eupatoria, Fructus Ligustri Lucidi, Spatholobus Suberectus Dunn, Sophorae Flavescentis Radix* |
| 20 | [9064](http://pubchem.ncbi.nlm.nih.gov/summary/summary.cgi?cid=9064) | [(+)-catechin](https://tcmsp-e.com/molecule.php?qn=492) | 290.29 | 54.83 | 0.24 | *Agrimonia Eupatoria , Spatholobus Suberectus Dunn* |
| 21 | [5281855](https://pubchem.ncbi.nlm.nih.gov/compound/5281855) | [ellagic acid](https://tcmsp-e.com/molecule.php?qn=1002) | 302.19 | 43.06 | 0.43 | *Agrimonia Eupatoria* |
| 22 | [5280794](http://pubchem.ncbi.nlm.nih.gov/summary/summary.cgi?cid=5280794) | [Stigmasterol](https://tcmsp-e.com/molecule.php?qn=449) | 412.77 | 43.83 | 0.76 | *Coicis Semen, Spatholobus Suberectus Dunn, Hedyotis Diffusae Herba, Radix Clematidis* |
| 23 | [12303645](http://pubchem.ncbi.nlm.nih.gov/summary/summary.cgi?cid=12303645) | [sitosterol](https://tcmsp-e.com/molecule.php?qn=359) | 414.79 | 36.91 | 0.75 | *Coicis Semen, Figwort Root* |
| 24 | [5997](http://pubchem.ncbi.nlm.nih.gov/summary/summary.cgi?cid=5997) | [CLR](https://tcmsp-e.com/molecule.php?qn=953) | 386.73 | 37.87 | 0.68 | *Coicis Semen* |
| 25 | [9548595](http://pubchem.ncbi.nlm.nih.gov/summary/summary.cgi?cid=9548595) | [Sitosterol alpha1](https://tcmsp-e.com/molecule.php?qn=1323) | 426.8 | 43.28 | 0.78 | *Coicis Semen* |
| 26 | [5282184](http://pubchem.ncbi.nlm.nih.gov/summary/summary.cgi?cid=5282184) | [Mandenol](https://tcmsp-e.com/molecule.php?qn=1494) | 308.56 | 42 | 0.19 | *Coicis Semen* |
| 27 | [11975273](http://pubchem.ncbi.nlm.nih.gov/summary/summary.cgi?cid=11975273) | [(6Z,10E,14E,18E)-2,6,10,15,19,23-hexamethyltetracosa-2,6,10,14,18,22-hexaene](https://tcmsp-e.com/molecule.php?qn=2372) | 410.8 | 33.55 | 0.42 | *Coicis Semen* |
| 28 | [11451146](http://pubchem.ncbi.nlm.nih.gov/summary/summary.cgi?cid=11451146) | [[(2R)-2,3-dihydroxypropyl] (Z)-octadec-9-enoate](https://tcmsp-e.com/molecule.php?qn=2882) | 356.61 | 34.13 | 0.3 | *Coicis Semen* |
| 29 | [46173943](https://pubchem.ncbi.nlm.nih.gov/compound/46173943) | [Coixenolide](https://tcmsp-e.com/molecule.php?qn=8118) | 591 | 32.4 | 0.43 | *Coicis Semen* |
| 30 | [5319879](http://pubchem.ncbi.nlm.nih.gov/summary/summary.cgi?cid=5319879) | [2-Monoolein](https://tcmsp-e.com/molecule.php?qn=8121) | 356.61 | 34.23 | 0.29 | *Coicis Semen* |
| 31 | [10181133](http://pubchem.ncbi.nlm.nih.gov/summary/summary.cgi?cid=10181133) | [Cerevisterol](https://tcmsp-e.com/molecule.php?qn=279) | 430.74 | 37.96 | 0.77 | *Polyporus Umbellatus* |
| 32 | [5283628](http://pubchem.ncbi.nlm.nih.gov/summary/summary.cgi?cid=5283628) | [ergosta-7,22E-dien-3beta-ol](https://tcmsp-e.com/molecule.php?qn=282) | 398.74 | 43.51 | 0.72 | *Polyporus Umbellatus* |
| 33 | [14236575](http://pubchem.ncbi.nlm.nih.gov/summary/summary.cgi?cid=14236575) | [(22e,24r)-ergosta-6-en-3beta,5alpha,6beta-triol](https://tcmsp-e.com/molecule.php?qn=796) | 432.76 | 30.2 | 0.76 | *Polyporus Umbellatus* |
| 34 | [99511](https://pubchem.ncbi.nlm.nih.gov/compound/99511) | [ergosta-7,22-dien-3-one](https://tcmsp-e.com/molecule.php?qn=816) | 396.6 | 44.88 | 0.72 | *Polyporus Umbellatus* |
| 35 | [247705](https://pubchem.ncbi.nlm.nih.gov/compound/247705) | [ergosta-5,7,22-trien-3-ol](https://tcmsp-e.com/molecule.php?qn=817) | 396.6 | 46.18 | 0.72 | *Polyporus Umbellatus* |
| 36 | [44575602](http://pubchem.ncbi.nlm.nih.gov/summary/summary.cgi?cid=44575602) | [polyporusterone E](https://tcmsp-e.com/molecule.php?qn=820) | 460.72 | 45.71 | 0.85 | *Polyporus Umbellatus* |
| 37 | [5351516](https://pubchem.ncbi.nlm.nih.gov/compound/5351516) | [Peroxyergosterol](https://tcmsp-e.com/molecule.php?qn=11169) | 428.6 | 44.39 | 0.82 | *Polyporus Umbellatus* |
| 38 | [222284](http://pubchem.ncbi.nlm.nih.gov/summary/summary.cgi?cid=222284) | [beta-sitosterol](https://tcmsp-e.com/molecule.php?qn=358) | 414.79 | 36.91 | 0.75 | *Figwort Root, Fructus Ligustri Lucidi, Spatholobus Suberectus Dunn, Hedyotis Diffusae Herba, Radix Clematidis* |
| 39 | [94162](https://pubchem.ncbi.nlm.nih.gov/compound/94162) | [sugiol](https://tcmsp-e.com/molecule.php?qn=2222) | 300.4 | 36.11 | 0.28 | *Figwort Root* |
| 40 | [6918700](https://pubchem.ncbi.nlm.nih.gov/compound/6918700) | [scropolioside D](https://tcmsp-e.com/molecule.php?qn=7659) | 722.7 | 36.62 | 0.4 | *Figwort Root* |
| 41 | [5281542](https://pubchem.ncbi.nlm.nih.gov/compound/5281542) | [harpagoside_qt](https://tcmsp-e.com/molecule.php?qn=7662) | 332.38 | 122.87 | 0.32 | *Figwort Root* |
| 42 | [439533](http://pubchem.ncbi.nlm.nih.gov/summary/summary.cgi?cid=439533) | [taxifolin](https://tcmsp-e.com/molecule.php?qn=4576) | 304.27 | 57.84 | 0.27 | *Fructus Ligustri Lucidi* |
| 43 | [10531060](https://pubchem.ncbi.nlm.nih.gov/compound/10531060) | [Lucidumoside D](https://tcmsp-e.com/molecule.php?qn=5146) | 568.6 | 48.87 | 0.71 | *Fructus Ligustri Lucidi* |
| 44 | [440735](http://pubchem.ncbi.nlm.nih.gov/summary/summary.cgi?cid=440735) | [eriodictyol](https://tcmsp-e.com/molecule.php?qn=5190) | 288.27 | 71.79 | 0.24 | *Fructus Ligustri Lucidi* |
| 45 | [138756681](https://pubchem.ncbi.nlm.nih.gov/compound/138756681) | [Lucidusculine](https://tcmsp-e.com/molecule.php?qn=5209) | 401.5 | 30.11 | 0.75 | *Fructus Ligustri Lucidi* |
| 46 | [94348](https://pubchem.ncbi.nlm.nih.gov/compound/94348) | [Olitoriside](https://tcmsp-e.com/molecule.php?qn=5211) | 696.8 | 65.45 | 0.23 | *Fructus Ligustri Lucidi* |
| 47 | [5319771](http://pubchem.ncbi.nlm.nih.gov/summary/summary.cgi?cid=5319771) | [8-o-Methylreyusi](https://tcmsp-e.com/molecule.php?qn=468) | 298.31 | 70.32 | 0.27 | *Spatholobus Suberectus Dunn* |
| 48 | [160608](http://pubchem.ncbi.nlm.nih.gov/summary/summary.cgi?cid=160608) | [3-Hydroxystigmast-5-en-7-one](https://tcmsp-e.com/molecule.php?qn=469) | 428.77 | 40.93 | 0.78 | *Spatholobus Suberectus Dunn* |
| 49 | [14018803](http://pubchem.ncbi.nlm.nih.gov/summary/summary.cgi?cid=14018803) | 8-C-α-L-arabinosylluteolin | 418.38 | 35.54 | 0.66 | *Spatholobus Suberectus Dunn* |
| 50 | [10207](http://pubchem.ncbi.nlm.nih.gov/summary/summary.cgi?cid=10207) | [aloe-emodin](https://tcmsp-e.com/molecule.php?qn=471) | 270.25 | 83.38 | 0.24 | *Spatholobus Suberectus Dunn* |
| 51 | [6440659](http://pubchem.ncbi.nlm.nih.gov/summary/summary.cgi?cid=6440659) | [(Z)-3-(4-hydroxy-3-methoxy-phenyl)-N-[2-(4-hydroxyphenyl)ethyl]acrylamide](https://tcmsp-e.com/molecule.php?qn=483) | 313.38 | 118.35 | 0.26 | *Spatholobus Suberectus Dunn* |
| 52 | [441774](https://pubchem.ncbi.nlm.nih.gov/compound/441774) | [petunidin](https://tcmsp-e.com/molecule.php?qn=490) | 317.29 | 30.05 | 0.31 | *Spatholobus Suberectus Dunn* |
| 53 | [5318998](http://pubchem.ncbi.nlm.nih.gov/summary/summary.cgi?cid=5318998) | [licochalcone a](https://tcmsp-e.com/molecule.php?qn=497) | 338.43 | 40.79 | 0.29 | *Spatholobus Suberectus Dunn* |
| 54 | [177149](http://pubchem.ncbi.nlm.nih.gov/summary/summary.cgi?cid=177149) | [Vestitol](https://tcmsp-e.com/molecule.php?qn=500) | 272.32 | 74.66 | 0.21 | *Spatholobus Suberectus Dunn* |
| 55 | [5281706](http://pubchem.ncbi.nlm.nih.gov/summary/summary.cgi?cid=5281706) | [Cajinin](https://tcmsp-e.com/molecule.php?qn=502) | 300.28 | 68.8 | 0.27 | *Spatholobus Suberectus Dunn* |
| 56 | [5319322](https://pubchem.ncbi.nlm.nih.gov/compound/5319322) | [Medicagol](https://tcmsp-e.com/molecule.php?qn=503) | 296.24 | 57.49 | 0.6 | *Spatholobus Suberectus Dunn* |
| 57 | [644020](https://pubchem.ncbi.nlm.nih.gov/compound/644020) | [Lupinidine](https://tcmsp-e.com/molecule.php?qn=506) | 234.38 | 61.89 | 0.21 | *Spatholobus Suberectus Dunn* |
| 58 | [5281805](http://pubchem.ncbi.nlm.nih.gov/summary/summary.cgi?cid=5281805) | [Psi-Baptigenin](https://tcmsp-e.com/molecule.php?qn=507) | 282.26 | 70.12 | 0.31 | *Spatholobus Suberectus Dunn* |
| 59 | [5281330](http://pubchem.ncbi.nlm.nih.gov/summary/summary.cgi?cid=5281330) | [Poriferasterol](https://tcmsp-e.com/molecule.php?qn=1659) | 412.77 | 43.83 | 0.76 | *Hedyotis Diffusae Herba* |
| 60 | [91572](https://pubchem.ncbi.nlm.nih.gov/compound/91572) | [Phaseolin](https://tcmsp-e.com/molecule.php?qn=456) | 322.38 | 78.2 | 0.73 | *Sophorae Flavescentis Radix* |
| 61 | [667495](http://pubchem.ncbi.nlm.nih.gov/summary/summary.cgi?cid=667495) | [(2R)-5,7-dihydroxy-2-(4-hydroxyphenyl)chroman-4-one](https://tcmsp-e.com/molecule.php?qn=1040) | 272.27 | 42.36 | 0.21 | *Sophorae Flavescentis Radix* |
| 62 | [91510](https://pubchem.ncbi.nlm.nih.gov/compound/91510) | [Inermine](https://tcmsp-e.com/molecule.php?qn=1484) | 284.26 | 75.18 | 0.54 | *Sophorae Flavescentis Radix* |
| 63 | [441298](http://pubchem.ncbi.nlm.nih.gov/summary/summary.cgi?cid=441298) | [hyperforin](https://tcmsp-e.com/molecule.php?qn=3347) | 536.87 | 44.03 | 0.6 | *Sophorae Flavescentis Radix* |
| 64 | [5318624](http://pubchem.ncbi.nlm.nih.gov/summary/summary.cgi?cid=5318624) | [8-Isopentenyl-kaempferol](https://tcmsp-e.com/molecule.php?qn=3542) | 354.38 | 38.04 | 0.39 | *Sophorae Flavescentis Radix* |
| 65 | [115269](https://pubchem.ncbi.nlm.nih.gov/compound/115269) | [sophocarpine](https://tcmsp-e.com/molecule.php?qn=3627) | 246.35 | 64.26 | 0.25 | *Sophorae Flavescentis Radix* |
| 66 | [91510](https://pubchem.ncbi.nlm.nih.gov/compound/91510) | [Inermin](https://tcmsp-e.com/molecule.php?qn=3648) | 284.26 | 65.83 | 0.54 | *Sophorae Flavescentis Radix* |
| 67 | [5281814](http://pubchem.ncbi.nlm.nih.gov/summary/summary.cgi?cid=5281814) | [Wighteone](https://tcmsp-e.com/molecule.php?qn=3673) | 338.38 | 42.8 | 0.36 | *Sophorae Flavescentis Radix* |
| 68 | [169014](https://pubchem.ncbi.nlm.nih.gov/compound/169014) | [Sophoramine](https://tcmsp-e.com/molecule.php?qn=3676) | 244.33 | 42.16 | 0.25 | *Sophorae Flavescentis Radix* |
| 69 | [165549](https://pubchem.ncbi.nlm.nih.gov/compound/165549) | [sophoridine](https://tcmsp-e.com/molecule.php?qn=3680) | 248.36 | 60.07 | 0.25 | *Sophorae Flavescentis Radix* |
| 70 | [443758](http://pubchem.ncbi.nlm.nih.gov/summary/summary.cgi?cid=443758) | [cis-Dihydroquercetin](https://tcmsp-e.com/molecule.php?qn=4580) | 304.27 | 66.44 | 0.27 | *Sophorae Flavescentis Radix* |
| 71 | [676152](http://pubchem.ncbi.nlm.nih.gov/summary/summary.cgi?cid=676152) | [5,7-dihydroxy-2-(3-hydroxy-4-methoxyphenyl)chroman-4-one](https://tcmsp-e.com/molecule.php?qn=5100) | 302.3 | 47.74 | 0.27 | *Sophorae Flavescentis Radix* |
| 72 | [91466](https://pubchem.ncbi.nlm.nih.gov/compound/91466) | [matrine](https://tcmsp-e.com/molecule.php?qn=5944) | 248.36 | 63.77 | 0.25 | *Sophorae Flavescentis Radix* |
| 73 | [3073381](https://pubchem.ncbi.nlm.nih.gov/compound/3073381) | [(+)-14alpha-hydroxymatrine](https://tcmsp-e.com/molecule.php?qn=6561) | 264.36 | 35.73 | 0.29 | *Sophorae Flavescentis Radix* |
| 74 | [15385684](https://pubchem.ncbi.nlm.nih.gov/compound/15385684) | [(+)-9alpha-hydroxymatrine](https://tcmsp-e.com/molecule.php?qn=6563) | 264.36 | 32.04 | 0.29 | *Sophorae Flavescentis Radix* |
| 75 | [7000681](https://pubchem.ncbi.nlm.nih.gov/compound/7000681) | [(+)-allomatrine](https://tcmsp-e.com/molecule.php?qn=6564) | 248.36 | 58.87 | 0.25 | *Sophorae Flavescentis Radix* |
| 76 | [3041752](https://pubchem.ncbi.nlm.nih.gov/compound/3041752) | [(+)-lehmannine](https://tcmsp-e.com/molecule.php?qn=6566) | 246.35 | 58.34 | 0.25 | *Sophorae Flavescentis Radix* |
| 77 | [22295472](https://pubchem.ncbi.nlm.nih.gov/compound/22295472) | [(-)-14beta-hydroxymatrine](https://tcmsp-e.com/molecule.php?qn=6569) | 264.41 | 37.26 | 0.29 | *Sophorae Flavescentis Radix* |
| 78 | [4113](https://pubchem.ncbi.nlm.nih.gov/compound/4113) | [(-)-9alpha-hydroxysophoramine](https://tcmsp-e.com/molecule.php?qn=6570) | 262.39 | 35.23 | 0.29 | *Sophorae Flavescentis Radix* |
| 79 | [54605823](https://pubchem.ncbi.nlm.nih.gov/compound/54605823) | [anagyrine](https://tcmsp-e.com/molecule.php?qn=6571) | 325.24 | 62.01 | 0.24 | *Sophorae Flavescentis Radix* |
| 80 | [162807](https://pubchem.ncbi.nlm.nih.gov/compound/162807) | [Glyceollin](https://tcmsp-e.com/molecule.php?qn=6596) | 338.4 | 97.27 | 0.76 | *Sophorae Flavescentis Radix* |
| 81 | [9928523](http://pubchem.ncbi.nlm.nih.gov/summary/summary.cgi?cid=9928523) | [(2S)-7-hydroxy-2-(4-hydroxyphenyl)-5-methoxy-8-(3-methylbut-2-enyl)chroman-4-one](https://tcmsp-e.com/molecule.php?qn=6604) | 354.43 | 48.09 | 0.39 | *Sophorae Flavescentis Radix* |
| 82 | [5318889](https://pubchem.ncbi.nlm.nih.gov/compound/5318889) | [kushenin](https://tcmsp-e.com/molecule.php?qn=6613) | 286.28 | 47.62 | 0.38 | *Sophorae Flavescentis Radix* |
| 83 | [44257224](https://pubchem.ncbi.nlm.nih.gov/compound/44257224) | [kushenol O](https://tcmsp-e.com/molecule.php?qn=6622) | 562.57 | 42.41 | 0.76 | *Sophorae Flavescentis Radix* |
| 84 | [25201713](https://pubchem.ncbi.nlm.nih.gov/compound/25201713) | [leachianone,g](https://tcmsp-e.com/molecule.php?qn=6626) | 356.4 | 60.97 | 0.4 | *Sophorae Flavescentis Radix* |
| 85 | [3041752](https://pubchem.ncbi.nlm.nih.gov/compound/3041752) | [Lehmanine](https://tcmsp-e.com/molecule.php?qn=6627) | 246.35 | 62.23 | 0.25 | *Sophorae Flavescentis Radix* |
| 86 | [91471](https://pubchem.ncbi.nlm.nih.gov/compound/91471) | [(+)-Lupanine](https://tcmsp-e.com/molecule.php?qn=6628) | 248.36 | 52.71 | 0.24 | *Sophorae Flavescentis Radix* |
| 87 | [5481970](http://pubchem.ncbi.nlm.nih.gov/summary/summary.cgi?cid=5481970) | [Norartocarpetin](https://tcmsp-e.com/molecule.php?qn=6630) | 286.25 | 54.93 | 0.24 | *Sophorae Flavescentis Radix* |
| 88 | [23724669](https://pubchem.ncbi.nlm.nih.gov/compound/23724669) | [(-)-Maackiain-3-O-glucosyl-6'-O-malonate](https://tcmsp-e.com/molecule.php?qn=6650) | 532.4 | 48.69 | 0.52 | *Sophorae Flavescentis Radix* |
| 89 | [442827](https://pubchem.ncbi.nlm.nih.gov/compound/442827) | [trifolrhizin](https://tcmsp-e.com/molecule.php?qn=6652) | 446.4 | 48.53 | 0.74 | *Sophorae Flavescentis Radix* |
| 90 | [11975273](http://pubchem.ncbi.nlm.nih.gov/summary/summary.cgi?cid=11975273) | [(6Z,10E,14E,18E)-2,6,10,15,19,23-hexamethyltetracosa-2,6,10,14,18,22-hexaene](https://tcmsp-e.com/molecule.php?qn=2372) | 410.8 | 33.55 | 0.42 | *Radix Clematidis* |
| 91 | [21155963](http://pubchem.ncbi.nlm.nih.gov/summary/summary.cgi?cid=21155963) | [Embinin](https://tcmsp-e.com/molecule.php?qn=5598) | 606.63 | 33.91 | 0.73 | *Radix Clematidis* |
| 92 | [19284](http://pubchem.ncbi.nlm.nih.gov/summary/summary.cgi?cid=19284) | [Heptyl phthalate](https://tcmsp-e.com/molecule.php?qn=5603) | 362.56 | 42.26 | 0.31 | *Radix Clematidis* |

**Supplementary Table 3 Common targets**

| **Number** | **Gene Name** | **Number** | **Gene Name** | **Number** | **Gene Name** | **Number** | **Gene Name** | **Number** | **Gene Name** | **Number** | **Gene Name** |
| --- | --- | --- | --- | --- | --- | --- | --- | --- | --- | --- | --- |
| 1 | ABCB1 | 71 | CCNB2 | 141 | ERBB4 | 211 | IGFBP3 | 281 | NOS2 | 351 | PTPA |
| 2 | ABCC1 | 72 | CCND1 | 142 | ERCC5 | 212 | IKBKB | 282 | NOS3 | 352 | PTPN1 |
| 3 | ABCG2 | 73 | CCND2 | 143 | ESR | 213 | IKBKE | 283 | NOX1 | 353 | PTPN11 |
| 4 | ABL1 | 74 | CCNE1 | 144 | ESR1 | 214 | IL2 | 284 | NOX4 | 354 | PTPN6 |
| 5 | ACE | 75 | CCNH | 145 | ESR2 | 215 | IL6 | 285 | NQO2 | 355 | PTPRC |
| 6 | ACHE | 76 | CCR4 | 146 | EZR | 216 | IL6ST | 286 | NR1H2 | 356 | RAC1 |
| 7 | ACKR3 | 77 | CD81 | 147 | F2 | 217 | INCENP | 287 | NR1H3 | 357 | RAF1 |
| 8 | ACP1 | 78 | CDC42 | 148 | F2R | 218 | INSR | 288 | NR1I2 | 358 | RARA |
| 9 | ADAM10 | 79 | CDK1 | 149 | F2RL1 | 219 | ITGB3 | 289 | NR1I3 | 359 | RARB |
| 10 | ADAMTS4 | 80 | CDK2 | 150 | F3 | 220 | ITK | 290 | NR3C1 | 360 | RELA |
| 11 | ADAMTS5 | 81 | CDK4 | 151 | FABP1 | 221 | JAK1 | 291 | NR3C2 | 361 | REN |
| 12 | ADH1A | 82 | CDK5 | 152 | FABP3 | 222 | JAK2 | 292 | NTRK1 | 362 | RET |
| 13 | ADH1C | 83 | CDK6 | 153 | FABP5 | 223 | JAK3 | 293 | NUAK1 | 363 | RIPK2 |
| 14 | ADORA1 | 84 | CDK7 | 154 | FASN | 224 | JUN | 294 | ODC1 | 364 | ROCK1 |
| 15 | ADORA2A | 85 | CDK9 | 155 | FEN1 | 225 | KCNH2 | 295 | OPRD1 | 365 | ROCK2 |
| 16 | ADRA1A | 86 | CFTR | 156 | FGF2 | 226 | KCNMA1 | 296 | OPRM1 | 366 | RORA |
| 17 | ADRA1B | 87 | CHEK1 | 157 | FGFR1 | 227 | KDM1A | 297 | P2RX7 | 367 | RORC |
| 18 | ADRA1D | 88 | CHEK2 | 158 | FGFR3 | 228 | KDR | 298 | PABPC1 | 368 | RXRA |
| 19 | ADRA2B | 89 | CHRM2 | 159 | FLT1 | 229 | KIF11 | 299 | PAK1 | 369 | S1PR1 |
| 20 | ADRB2 | 90 | CHRM3 | 160 | FLT3 | 230 | KISS1R | 300 | PARP1 | 370 | SCN9A |
| 21 | AGTR1 | 91 | CHRNA3 | 161 | FLT4 | 231 | KIT | 301 | PCNA | 371 | SELE |
| 22 | AHR | 92 | CHRNA4 | 162 | FOS | 232 | LGALS3 | 302 | PCSK7 | 372 | SERPINE1 |
| 23 | AKR1A1 | 93 | CHRNA5 | 163 | FPR1 | 233 | LGALS7 | 303 | PDE4D | 373 | SGK1 |
| 24 | AKR1B1 | 94 | CHRNB2 | 164 | FPR2 | 234 | LGALS9 | 304 | PDGFRA | 374 | SHBG |
| 25 | AKR1B10 | 95 | CLK2 | 165 | FUT4 | 235 | LIMK1 | 305 | PDGFRB | 375 | SHH |
| 26 | AKR1C3 | 96 | CMA1 | 166 | GLI1 | 236 | LNPEP | 306 | PDK1 | 376 | SIRT1 |
| 27 | AKT1 | 97 | COMT | 167 | GLI2 | 237 | LPAR1 | 307 | PDPK1 | 377 | SIRT2 |
| 28 | AKT2 | 98 | COX1 | 168 | GLO1 | 238 | LPAR2 | 308 | PFKFB3 | 378 | SIRT3 |
| 29 | ALDH2 | 99 | COX2 | 169 | GLUL | 239 | LYN | 309 | PGR | 379 | SLC16A1 |
| 30 | ALK | 100 | CPT1A | 170 | GPR35 | 240 | MAP2K1 | 310 | PIK3CA | 380 | SMO |
| 31 | ALOX12 | 101 | CREBBP | 171 | GRIK2 | 241 | MAP3K20 | 311 | PIK3CB | 381 | SOAT1 |
| 32 | ALOX15 | 102 | CSF1R | 172 | GRIN2B | 242 | MAP3K8 | 312 | PIK3CD | 382 | SRC |
| 33 | ALOX5 | 103 | CSK | 173 | GRK2 | 243 | MAP4K4 | 313 | PIK3CG | 383 | ST3GAL3 |
| 34 | ALPG | 104 | CSNK2A1 | 174 | GSK3B | 244 | MAPK1 | 314 | PIK3R1 | 384 | ST6GAL1 |
| 35 | ANPEP | 105 | CTSB | 175 | GSTM2 | 245 | MAPK14 | 315 | PIM1 | 385 | STAT1 |
| 36 | APEX1 | 106 | CTSD | 176 | GSTP1 | 246 | MAPK3 | 316 | PIM2 | 386 | STAT3 |
| 37 | APOBEC3A | 107 | CTSF | 177 | GUSB | 247 | MAPK8 | 317 | PIM3 | 387 | STAT6 |
| 38 | APP | 108 | CTSK | 178 | HCK | 248 | MAPK9 | 318 | PLA2B | 388 | STS |
| 39 | AR | 109 | CXCR1 | 179 | HCRTR1 | 249 | MAPT | 319 | PLA2G2A | 389 | SYK |
| 40 | ATP12A | 110 | CXCR2 | 180 | HDAC1 | 250 | MBD2 | 320 | PLA2L | 390 | TACR1 |
| 41 | ATP4A | 111 | CXCR3 | 181 | HDAC2 | 251 | MCL1 | 321 | PLAT | 391 | TBK1 |
| 42 | ATP4B | 112 | CYP19A1 | 182 | HDAC3 | 252 | MDM2 | 322 | PLAU | 392 | TEK |
| 43 | AURKA | 113 | CYP1A1 | 183 | HDAC4 | 253 | MDM4 | 323 | PLG | 393 | TERT |
| 44 | AURKB | 114 | CYP1A2 | 184 | HDAC5 | 254 | MELK | 324 | PLK1 | 394 | TGFBR1 |
| 45 | AXL | 115 | CYP2C19 | 185 | HDAC6 | 255 | MERTK | 325 | PLK3 | 395 | TGFBR2 |
| 46 | BCHE | 116 | CYP2D6 | 186 | HDAC7 | 256 | MET | 326 | PLK4 | 396 | TGM2 |
| 47 | BCL2 | 117 | CYP3A4 | 187 | HDAC8 | 257 | MGMT | 327 | PNMT | 397 | THRA |
| 48 | BCL2A1 | 118 | DAPK1 | 188 | HDAC9 | 258 | MIF | 328 | POLB | 398 | THRB |
| 49 | BCL2L1 | 119 | DNMT3A | 189 | HIF1A | 259 | MME | 329 | PON1 | 399 | TKT |
| 50 | BDKRB2 | 120 | DPP4 | 190 | HMGCR | 260 | MMP1 | 330 | PPARA | 400 | TLR9 |
| 51 | BIRC5 | 121 | DRD2 | 191 | HMOX1 | 261 | MMP10 | 331 | PPARD | 401 | TNF |
| 52 | BMP1 | 122 | EBP | 192 | HNF4A | 262 | MMP12 | 332 | PPARG | 402 | TNKS |
| 53 | BMX | 123 | ECE1 | 193 | HPGDS | 263 | MMP13 | 333 | PPIA | 403 | TOP2A |
| 54 | BRAF | 124 | EDNRA | 194 | HPSE | 264 | MMP14 | 334 | PPP1CA | 404 | TP53 |
| 55 | BRD4 | 125 | EDNRB | 195 | HRAS | 265 | MMP2 | 335 | PPP2CA | 405 | TRIM24 |
| 56 | BTK | 126 | EGFR | 196 | HRH2 | 266 | MMP25 | 336 | PRKCA | 406 | TTK |
| 57 | C5AR1 | 127 | EIF4A1 | 197 | HSD11B2 | 267 | MMP3 | 337 | PRKCB | 407 | TTR |
| 58 | CA9 | 128 | EP300 | 198 | HSD17B1 | 268 | MMP7 | 338 | PRKCH | 408 | TUBB3 |
| 59 | CAPN1 | 129 | EPAS1 | 199 | HSD17B2 | 269 | MMP9 | 339 | PRKCZ | 409 | TYMP |
| 60 | CASP1 | 130 | EPHA1 | 200 | HSD17B7 | 270 | MPEG1 | 340 | PRKDC | 410 | TYMS |
| 61 | CASP3 | 131 | EPHA2 | 201 | HSP90AA1 | 271 | MPO | 341 | PRMT1 | 411 | UBA2 |
| 62 | CASP7 | 132 | EPHA3 | 202 | HSP90AB1 | 272 | MST1R | 342 | PSEN1 | 412 | UGCG |
| 63 | CASP8 | 133 | EPHA5 | 203 | HSP90B1 | 273 | MTOR | 343 | PSEN2 | 413 | VCP |
| 64 | CASP9 | 134 | EPHA8 | 204 | HSPA1A | 274 | MYLK | 344 | PTGDR2 | 414 | VDR |
| 65 | CASR | 135 | EPHB1 | 205 | HSPA5 | 275 | NAMPT | 345 | PTGER3 | 415 | VEGFA |
| 66 | CBFB | 136 | EPHB2 | 206 | HSPA8 | 276 | NAT1 | 346 | PTGER4 | 416 | WEE1 |
| 67 | CBR1 | 137 | EPHB3 | 207 | ICAM1 | 277 | NEK2 | 347 | PTGS1 | 417 | XDH |
| 68 | CCKBR | 138 | EPHB4 | 208 | IDH1 | 278 | NET1 | 348 | PTGS2 | 418 | XIAP |
| 69 | CCNA2 | 139 | EPHX1 | 209 | IDO1 | 279 | NLRP3 | 349 | PTK2 | 419 | XPO1 |
| 70 | CCNB1 | 140 | ERBB2 | 210 | IGF1R | 280 | NOS1 | 350 | PTK6 | 420 | YES1 |

**Supplementary Table 4 The significantly enrichment potential term of GO function analysis**

| **Category** | **GO ID** | **GO Term** | **Count** | **P-value** | **Genes** |
| --- | --- | --- | --- | --- | --- |
| Biological Process | GO:0006468 | protein phosphorylation | 88 | 1.34E-54 | RET, APP, GSK3B, PIK3CD, PIK3CG, MYLK, IKBKB, EDNRA, TBK1, CCND1, CHEK2, CHEK1, PIM1, AKT1, PIM3, NEK2, MAP3K8, PIM2, JAK2, JAK3, IKBKE, JAK1, PDK1, MAP2K1, PRKCH, CSNK2A1, SYK, PDPK1, PRKCB, DAPK1, IGFBP3, ADAM10, PRKCA, TGFBR1, TGFBR2, HCK, MELK, CCNE1, INCENP, RARA, BTK, BIRC5, RAF1, SGK1, ROCK1, ROCK2, PRKDC, PIK3R1, PRKCZ, AURKB, AURKA, CDC42, MAPK9, NUAK1, PAK1, MAPK8, CCNB1, GRK2, ERBB2, ABL1, MAP3K20, MAPK1, CSK, MAPK3, MAP4K4, NTRK1, LYN, PLK4, PLK3, INSR, LIMK1, PLK1, BRAF, PTK6, BMX, MERTK, MTOR, PTK2, CLK2, CDK9, P2RX7, CDK7, CDK6, CDK5, CDK4, CDK2, CDK1, FGFR1 |
| Biological Process | GO:0045944 | positive regulation of transcription from RNA polymerase II promoter | 85 | 3.51E-22 | TOP2A, APP, THRB, THRA, KDM1A, CD81, RORA, AHR, GLI1, NR3C1, FGF2, TNF, GLI2, IKBKB, SHH, TBK1, HNF4A, NAMPT, AKT1, EP300, NOS1, JAK2, HRAS, PARP1, RIPK2, FOS, SIRT1, SIRT2, MMP12, AR, SMO, RARA, TLR9, RARB, PPARG, PGR, RAF1, PPARA, MET, TP53, PPARD, HDAC4, HDAC5, HDAC2, HDAC3, CBFB, TNKS, PRKDC, EPAS1, HDAC1, NR1I3, NR1I2, ADRB2, PIK3R1, HDAC8, HIF1A, EGFR, RELA, RXRA, CXCR3, ABL1, S1PR1, NLRP3, STAT6, DRD2, BRD4, MAPK3, JUN, CREBBP, YES1, STAT1, VDR, NR1H2, STAT3, NR1H3, MAPK14, ESR1, ESR2, IL2, VEGFA, CDK9, IL6, CDK7, APEX1, F2RL1 |
| Biological Process | GO:0007165 | signal transduction | 85 | 6.12E-21 | RET, ALK, CHRM3, GSK3B, ITK, PIK3CD, PIK3CB, NR3C1, ADRA1A, FGF2, IGF1R, NR3C2, HPGDS, EDNRA, PLAU, ANPEP, AKT2, NAMPT, ADORA1, CASP1, AKT1, JAK2, HRAS, PDGFRB, CHRNB2, MAP2K1, PRKCH, CSNK2A1, RIPK2, PRKCB, DAPK1, PDE4D, TGFBR1, AR, KIT, RARA, RARB, PPARG, PGR, RAF1, MET, CSF1R, CHRNA3, ROCK1, CHRNA5, SRC, CHRNA4, EPAS1, NR1I3, NR1I2, C5AR1, FPR1, MST1R, PIK3R1, HIF1A, PRKCZ, EGFR, TYMP, GRK2, TTR, ERBB4, ERBB2, CXCR2, MAPK1, NLRP3, STAT6, LYN, CREBBP, STAT1, LIMK1, STAT3, BRAF, LNPEP, BMX, MAPK14, ESR1, ESR2, NET1, BMP1, CDK6, AXL, CDK4, CDK2, TEK, NOX1 |
| Biological Process | GO:0043066 | negative regulation of apoptotic process | 78 | 3.69E-42 | GSK3B, HSP90AB1, FLT4, AKR1B1, MPO, TNF, IGF1R, GLI2, IKBKB, SHH, EDNRB, CCND2, CASP3, AKT2, ADORA1, PIM1, KDR, AKT1, PIM3, PIM2, TGM2, PDGFRB, DAPK1, MIF, MMP9, SIRT1, TGFBR1, HCK, SMO, BIRC5, RAF1, IL6ST, TP53, PPARD, CSF1R, HDAC2, HDAC3, BCL2A1, SRC, PRKDC, HDAC1, GSTP1, GLO1, PSEN2, XIAP, PSEN1, PIK3R1, PRKCZ, EGFR, RELA, AURKA, HSP90B1, MAPK8, ERBB4, ERBB2, MCL1, MAP4K4, NTRK1, PLK3, HSPA5, MGMT, PLK1, BRAF, MTOR, IL2, PTK2, VEGFA, FABP1, IL6, AXL, BCL2, CDK1, MDM2, ERCC5, MDM4, TEK, BCL2L1, HSPA1A |
| Biological Process | GO:0008284 | positive regulation of cell proliferation | 70 | 1.61E-33 | FLT1, FLT3, CD81, FLT4, ADRA1D, GLI1, FGF2, IGF1R, SHH, EDNRB, CCND2, AKT2, NAMPT, KDR, AKT1, CAPN1, HRAS, PDGFRB, PDGFRA, CASR, CSNK2A1, PRMT1, F2R, ADAM10, MIF, F2, SIRT1, TGFBR1, TGFBR2, AR, HCK, CCKBR, KIT, RARA, BIRC5, IL6ST, EPHA1, HDAC4, CSF1R, HDAC2, HDAC1, ODC1, TTK, MST1R, PRKCZ, EGFR, RELA, DPP4, PAK1, ERBB4, CXCR3, ERBB2, CXCR2, LYN, INSR, AKR1C3, IL2, PTK2, VEGFA, IL6, CDK4, CDK2, BCL2, MDM2, PTPN6, HPSE, FGFR3, NOX1, FGFR1, BCL2L1 |
| Biological Process | GO:0000122 | negative regulation of transcription from RNA polymerase II promoter | 63 | 2.82E-14 | APP, THRB, THRA, KDM1A, RORC, NR3C1, TNF, GLI2, SHH, EDNRB, CCND1, EP300, PARP1, DNMT3A, SIRT1, SIRT2, MMP12, AR, SMO, CCNE1, RARA, RARB, PPARG, EZR, PPARA, TP53, PPARD, HDAC4, HDAC5, HDAC2, HDAC3, PCNA, CBFB, HDAC1, NR1I3, NR1I2, PSEN1, HDAC8, HDAC9, HDAC6, RELA, AURKB, HDAC7, RXRA, STAT6, PLK3, JUN, CREBBP, STAT1, VDR, NR1H2, PLK1, MBD2, STAT3, NR1H3, ESR1, ESR2, VEGFA, CDK6, CDK2, MDM2, MDM4, HSPA1A |
| Biological Process | GO:0006954 | inflammatory response | 57 | 1.48E-28 | PIK3CD, TNF, PIK3CG, POLB, IKBKB, MMP25, TBK1, SCN9A, ADORA1, BDKRB2, AKT1, RAC1, LGALS9, CCR4, RIPK2, F2R, FOS, MIF, TACR1, HCK, ADORA2A, KIT, AGTR1, TLR9, IDO1, EPHA2, HDAC4, PTGER4, CSF1R, HDAC5, C5AR1, PTGER3, ALOX15, FPR1, FPR2, PTGS2, HDAC9, PRKCZ, RELA, FUT4, PTGS1, CXCR3, CXCR2, NLRP3, NOS2, PLA2G2A, STAT3, SELE, MTOR, P2RX7, IL6, AXL, FASN, NOX4, F2RL1, HPSE, NOX1 |
| Biological Process | GO:0006915 | apoptotic process | 56 | 1.12E-19 | APP, AHR, NR3C1, ADRA1A, CASP9, CASP7, CASP8, CASP3, CHEK1, PIM1, CASP1, EP300, PIM3, PIM2, JAK2, LGALS7, CASR, PARP1, CSNK2A1, RIPK2, PRKCB, DAPK1, IGFBP3, MMP9, TGFBR1, TGFBR2, MELK, ADORA2A, SMO, RARB, BIRC5, RAF1, SGK1, MET, PPIA, TP53, PPARD, ROCK1, BCL2A1, PSEN1, AURKA, PPP2CA, PAK1, CXCR3, MAPK1, NLRP3, MCL1, MAPK3, PLK3, JUN, BMX, MAPK14, BCL2, CDK1, MDM2, BCL2L1 |
| Biological Process | GO:0045893 | positive regulation of transcription, DNA-templated | 55 | 1.51E-15 | RET, RORC, RORA, AHR, GLI1, FGF2, TNF, GLI2, IKBKB, SHH, CHEK2, HNF4A, PIM1, TRIM24, AKT1, EP300, PIM2, NOS1, KCNH2, MAP2K1, PRKCB, F2R, FOS, TGFBR1, CCNA2, AR, RARA, PPARG, PPARA, TP53, PPARD, HDAC4, HDAC2, SRC, HDAC1, NR1I2, PSEN1, HIF1A, EGFR, RELA, RXRA, ERBB4, BRD4, JUN, CREBBP, STAT1, INSR, NR1H2, MBD2, STAT3, NR1H3, ESR1, ESR2, IL6, CDK2 |
| Biological Process | GO:0046777 | protein autophosphorylation | 54 | 2.02E-45 | ALK, GSK3B, FLT1, FLT3, FLT4, IGF1R, CHEK2, PIM1, KDR, TRIM24, AKT1, PIM3, NEK2, PIM2, JAK2, EPHB1, EPHB4, EPHB3, PDGFRB, PDGFRA, SYK, PDPK1, DAPK1, EPHA8, HCK, MELK, KIT, BTK, EPHA1, CSF1R, SRC, TTK, EGFR, AURKB, AURKA, PAK1, ERBB4, ERBB2, ABL1, MAP3K20, CSK, NTRK1, LYN, INSR, PTK6, BMX, MTOR, PTK2, CLK2, PTPRC, CDK5, TEK, FGFR3, FGFR1 |
| Biological Process | GO:0010628 | positive regulation of gene expression | 52 | 5.68E-20 | RET, APP, GSK3B, ITGB3, PIK3CD, PIK3CB, FGF2, TNF, SHH, TRIM24, AKT1, LGALS9, EPHB2, LGALS7, CASR, MAP2K1, F3, TGFBR1, AR, SMO, RARA, TLR9, PPARG, PGR, EZR, TP53, PPARD, ROCK1, ROCK2, HDAC1, NR1I2, PSEN1, HIF1A, RELA, MAPK9, MAPK8, ERBB2, MAPK1, MAPK3, NOS3, VDR, NR1H2, STAT3, BRAF, MAPK14, MTOR, VEGFA, P2RX7, IL6, CDK6, CDK1, HSPA1A |
| Biological Process | GO:0018108 | peptidyl-tyrosine phosphorylation | 51 | 1.02E-47 | RET, ITK, FLT1, FLT3, FLT4, KDR, EPHB2, JAK2, JAK3, EPHB1, EPHB4, EPHB3, PDGFRB, EPHA5, PDGFRA, MAP2K1, SYK, RIPK2, EPHA8, HCK, MELK, KIT, BTK, EPHA1, MET, EPHA3, EPHA2, CSF1R, SRC, TTK, MST1R, EGFR, ERBB4, ERBB2, ABL1, CSK, NTRK1, LYN, YES1, INSR, BMX, MERTK, PTK2, CLK2, WEE1, PTPRC, AXL, PTPN6, TEK, FGFR3, FGFR1 |
| Biological Process | GO:0035556 | intracellular signal transduction | 46 | 7.97E-18 | GSK3B, ITK, ROCK1, SRC, PSEN2, PSEN1, ADRA1B, ADRA1A, PRKCZ, MAPK9, PAK1, CASP7, MAPK8, NUAK1, AKT2, ERBB2, CHEK1, AKT1, HMOX1, MAPK1, RAC1, JAK2, DRD2, JAK3, JAK1, MAPK3, MAP4K4, LYN, PRKCH, SYK, PRKCB, DAPK1, PDPK1, PRKCA, BRAF, BMX, MAPK14, TGFBR1, NET1, MELK, KIT, BTK, PTPN6, RAF1, SGK1 |
| Biological Process | GO:0030335 | positive regulation of cell migration | 44 | 1.63E-25 | RET, CSF1R, FLT1, ROCK2, FLT4, PIK3CD, PIK3R1, GLI1, EGFR, IGF1R, MYLK, CDC42, PAK1, PLAU, AKT2, KDR, S1PR1, EPHB2, JAK2, HRAS, MAP4K4, LYN, PDGFRB, PDGFRA, MMP7, HSPA5, MMP2, INSR, F2R, STAT3, ADAM10, PRKCA, MMP9, F3, TGFBR1, PTK2, VEGFA, MMP14, SMO, KIT, F2RL1, HPSE, EPHA1, EPHA2 |
| Biological Process | GO:0043410 | positive regulation of MAPK cascade | 43 | 1.85E-33 | RET, ALK, APP, FLT1, ROCK1, ROCK2, CD81, FLT3, FLT4, LPAR1, LPAR2, ADRA1D, ADRB2, ADRA1B, FGF2, ADRA1A, TNF, IGF1R, CDC42, ERBB2, ADORA1, KDR, JAK2, HRAS, NTRK1, MAP2K1, EPHA8, IGFBP3, INSR, F2R, ADRA2B, TGFBR1, VEGFA, AR, IL6, PTPRC, KIT, TLR9, TEK, RAF1, FGFR3, PPIA, FGFR1 |
| Biological Process | GO:0070374 | positive regulation of ERK1 and ERK2 cascade | 43 | 3.61E-27 | CSF1R, APP, SRC, FLT4, ITGB3, C5AR1, ALOX15, FPR2, FGF2, ADRA1A, TNF, PRKCZ, EGFR, ICAM1, ERBB4, KDR, ABL1, LGALS9, DRD2, HRAS, MAPK3, PDGFRB, NTRK1, PDGFRA, JUN, CASR, MAP2K1, RIPK2, PLA2G2A, F2R, PRKCA, PTPN11, BRAF, MIF, OPRM1, HCRTR1, VEGFA, PTPRC, NOX4, F2RL1, ACKR3, TEK, FGFR3 |
| Biological Process | GO:0007186 | G-protein coupled receptor signaling pathway | 43 | 9.35E-06 | CHRM2, OPRD1, APP, CHRM3, PTGDR2, PTGER3, LPAR1, FPR1, LPAR2, ADRA1D, ECE1, PIK3CB, FPR2, ADRA1B, ADRA1A, PIK3CG, EDNRA, GRK2, CXCR1, CXCR3, ADORA1, KISS1R, S1PR1, BDKRB2, AKT1, JAK2, CCR4, TGM2, PDGFRB, CASR, GPR35, INSR, F2R, AKR1C3, ADRA2B, HCRTR1, SMO, CCKBR, AGTR1, F2RL1, ACKR3, PTPN6, PPARG |
| Biological Process | GO:0009410 | response to xenobiotic stimulus | 42 | 3.21E-24 | RET, HDAC4, HDAC2, ABCB1, HSP90AB1, SRC, AHR, TYMS, PTGS2, ADRA1A, TNF, HSD11B2, CCND1, CASP3, ABL1, HMOX1, CA9, DRD2, LYN, NTRK1, BCHE, ABCC1, JUN, CPT1A, HSP90AA1, ACE, MGMT, STAT1, MMP2, DNMT3A, FOS, TGFBR2, CDK9, P2RX7, ATP4A, ADORA2A, CDK4, APEX1, BCL2, CDK1, PPARG, TP53 |
| Biological Process | GO:0008285 | negative regulation of cell proliferation | 42 | 1.18E-14 | HDAC4, CSF1R, APP, PLG, PTGS2, FGF2, ADRA1A, ERBB4, HNF4A, ADORA1, TRIM24, CSK, PIM2, JAK2, DRD2, HRAS, LYN, NTRK1, BCHE, JUN, MAP2K1, NOS3, VDR, IGFBP3, F2R, STAT3, OPRM1, SIRT2, AR, IL6, FABP3, CDK6, ADORA2A, RARA, NOX4, RARB, ACKR3, PTPN6, MDM4, RAF1, TP53, HSPA1A |
| Biological Process | GO:0007169 | transmembrane receptor protein tyrosine kinase signaling pathway | 41 | 9.83E-35 | RET, ALK, CSF1R, FLT1, FLT3, SRC, FLT4, MST1R, PIK3CB, EGFR, IGF1R, ERBB4, ERBB2, KDR, BDKRB2, EPHB2, EPHB1, EPHB4, EPHB3, LYN, PDGFRB, NTRK1, EPHA5, PDGFRA, YES1, SYK, EPHA8, INSR, PTK6, MERTK, PTK2, HCK, AXL, KIT, TEK, EPHA1, MET, FGFR3, EPHA3, FGFR1, EPHA2 |
| Cellular Component | GO:0005886 | plasma membrane | 216 | 1.22E-28 | APP, SERPINE1, MYLK, ICAM1, LGALS3, EDNRA, EDNRB, STS, AKT2, ANPEP, KDR, AKT1, EPHB2, GLUL, EPHB1, EPHB4, EPHB3, EPHA5, PRKCH, CSNK2A1, DAPK1, PRKCB, EPHA8, PRKCA, MIF, AR, BTK, AGTR1, EZR, EPHA1, CFTR, EPHA3, EPHA2, ABCB1, FPR1, LPAR1, LPAR2, PIK3R1, FPR2, MST1R, PRKCZ, TERT, LYN, JUN, ACE, YES1, INSR, PTK6, BRAF, LNPEP, GRIN2B, PTK2, ATP4B, ATP4A, FASN, NOX4, REN, NOX1, RET, ACHE, ITK, PTGDR2, GRIK2, SHH, MMP25, CTSF, RAC1, JAK2, JAK3, HRAS, CCR4, SYK, MME, GPR35, TACR1, F2, F3, SIRT2, HCK, MMP14, CCKBR, KCNMA1, KIT, RARA, TLR9, MAPT, SGK1, PTGER4, OPRD1, ADH1C, ADH1A, PTGER3, EGFR, PPP2CA, PAK1, CXCR1, CXCR3, CXCR2, KISS1R, BCHE, SLC16A1, STAT3, PPP1CA, FABP5, AXL, F2RL1, HSPA1A, CHRM2, CHRM3, CD81, COMT, ATP12A, TNF, IGF1R, PLAU, SCN9A, ADORA1, PIM1, CAPN1, TGM2, PDGFRB, CHRNB2, KCNH2, PDGFRA, CASR, MAP2K1, F2R, ADAM10, MELK, SMO, RAF1, IL6ST, ABCG2, CSF1R, CHRNA3, CHRNA5, CHRNA4, GLO1, C5AR1, ALOX15, PSEN2, ALOX12, PSEN1, ADRB2, CYP2C19, HSP90B1, DPP4, GRK2, HRH2, DRD2, HSPA8, HSPA5, BMX, ESR1, SELE, CDK7, PTPRC, CDK5, MDM2, ALPG, FGFR3, FGFR1, ALK, GSK3B, HSP90AB1, FLT1, FLT3, ITGB3, FLT4, PIK3CD, ADRA1D, ECE1, PIK3CB, ADRA1B, ADRA1A, PIK3CG, BDKRB2, CASP1, CA9, NOS1, HSP90AA1, ABCC1, PDPK1, PDE4D, MMP2, OPRM1, ADRA2B, TGFBR1, TGFBR2, ADORA2A, PIK3CA, MET, HDAC3, ROCK1, SRC, ROCK2, PLG, HDAC8, CDC42, MAPK9, ERBB4, ERBB2, S1PR1, MAPK1, CSK, MAPK3, NTRK1, PTPN1, NOS2, NOS3, PLA2G2A, MERTK, HCRTR1, P2RX7, ACKR3, TEK |
| Cellular Component | GO:0005829 | cytosol | 215 | 5.07E-26 | PNMT, APP, EIF4A1, MYLK, LGALS3, TBK1, AKT2, CHEK1, NAMPT, TRIM24, AKT1, LGALS9, EPHB2, LGALS7, GLUL, EPHB1, EPHB4, EPHB3, PRKCH, CSNK2A1, PRKCB, PRKCA, MIF, AR, BTK, EZR, TP53, CFTR, EPHA3, IDO1, PRKDC, EPAS1, PIK3R1, HIF1A, PRKCZ, TERT, HMOX1, LYN, PLK4, CREBBP, GSTM2, YES1, PLK1, PTK6, BRAF, LNPEP, PTK2, NET1, AKR1B10, FASN, ITK, AKR1B1, KIF11, GLI1, GLI2, IKBKB, SHH, XPO1, RAC1, JAK2, JAK3, HRAS, IKBKE, JAK1, CBR1, PARP1, SYK, RIPK2, PRMT1, AKR1A1, FOS, SIRT1, SIRT2, HCK, MMP14, INCENP, RARA, PPARG, MAPT, PABPC1, SGK1, TKT, ADH1C, ADH1A, NR1I3, GSTP1, ODC1, XIAP, TYMS, RELA, TYMP, PPP2CA, PAK1, RXRA, HSD17B1, ALOX5, STAT6, XDH, NQO2, STAT1, NR1H2, STAT3, MBD2, AKR1C3, NR1H3, PPP1CA, FABP1, FABP3, FABP5, HSPA1A, AHR, COMT, NR3C1, ATP12A, NR3C2, HPGDS, CCND2, CCND1, PIM1, EP300, NEK2, MAP3K8, PIM3, CAPN1, TGM2, MAP2K1, CCNE1, PGR, RAF1, PPIA, VCP, PFKFB3, TNKS, GLO1, ALOX15, ALOX12, HSP90B1, CCNB2, CCNB1, GRK2, ABL1, MAP3K20, NLRP3, MCL1, HSPA8, HSPA5, VDR, IDH1, BMX, ESR1, CDK7, CDK6, NAT1, CDK5, CDK4, CDK2, BCL2, MDM2, CDK1, BCL2L1, FGFR1, GSK3B, HSP90AB1, THRA, FLT3, FLT4, PIK3CD, PIK3CB, ADRA1A, PIK3CG, CASP9, CASP7, CASP8, CASP3, CASP1, NOS1, ACP1, HSP90AA1, PDPK1, PDE4D, ADRA2B, TGFBR2, CCNA2, PIK3CA, BIRC5, HDAC4, HDAC5, HDAC3, BCL2A1, ROCK1, SRC, ROCK2, HDAC1, HDAC6, AURKB, AURKA, HDAC7, CDC42, MAPK9, MAPK8, ERBB4, ERBB2, MAPK1, CSK, MAPK3, PTPN1, NOS2, CMA1, NOS3, LIMK1, PTPN11, MAPK14, MTOR, PTPN6 |
| Cellular Component | GO:0005737 | cytoplasm | 212 | 1.79E-23 | APP, EIF4A1, MYLK, LGALS3, TBK1, CYP2D6, ANPEP, CHEK2, CHEK1, NAMPT, AKT1, LGALS9, GLUL, PRKCH, DAPK1, PRKCB, PRKCA, MIF, AR, BTK, EZR, TP53, CFTR, IDO1, ABCB1, EPAS1, FPR1, LPAR1, LPAR2, PIK3R1, FPR2, HIF1A, PRKCZ, NUAK1, TERT, LYN, PLK4, PLK3, CREBBP, GSTM2, PLK1, PTK6, LNPEP, GRIN2B, PTK2, FASN, REN, APOBEC3A, TOP2A, KIF11, GLI1, IKBKB, XPO1, HNF4A, RAC1, JAK2, JAK3, HRAS, IKBKE, JAK1, PARP1, SYK, MME, RIPK2, PRMT1, TACR1, SIRT1, SIRT2, MMP12, HCK, MMP14, RARA, TLR9, RARB, PPARG, MAPT, PABPC1, SGK1, NR1I3, GSTP1, ODC1, XIAP, PTGS2, TYMS, RELA, EGFR, PTGS1, PTPA, PAK1, HSD17B1, ALOX5, CXCR3, STAT6, MAP4K4, STAT1, NR1H2, STAT3, AKR1C3, NR1H3, VEGFA, PPP1CA, FABP5, APEX1, HSPA1A, PLAT, AHR, NR3C1, FGF2, IGF1R, HPGDS, CCND2, CCND1, TUBB3, PIM1, EP300, NEK2, PIM3, PIM2, CAPN1, PDGFRB, PDGFRA, CASR, ADAM10, MELK, CCNE1, PGR, RAF1, PPIA, VCP, TNKS, GLO1, ALOX12, CYP2C19, CCNB2, CCNB1, GRK2, ABL1, MAP3K20, NLRP3, MCL1, HSPA8, HSPA5, VDR, IDH1, ESR1, CDK7, CDK6, CDK5, CDK4, CDK2, BCL2, MDM2, CDK1, UBA2, BCL2L1, GSK3B, HSP90AB1, PIK3CD, PIK3CB, ADRA1B, ADRA1A, PIK3CG, POLB, CASP9, CASP7, CASP8, CASP3, CASP1, NOS1, ACP1, HSP90AA1, PDPK1, MMP3, DNMT3A, CCNA2, PIK3CA, BIRC5, HDAC4, HDAC5, HDAC2, HDAC3, BCL2A1, ROCK1, SRC, ROCK2, HDAC1, TTK, CYP3A4, HDAC8, HDAC9, HDAC6, HDAC7, CDC42, MAPK9, MAPK8, S1PR1, MAPK1, CSK, MAPK3, PTPN1, NOS2, CMA1, NOS3, LIMK1, PTPN11, MERTK, MAPK14, MTOR, P2RX7, WEE1, PTPN6, TEK |
| Cellular Component | GO:0005634 | nucleus | 207 | 4.60E-18 | APP, CCNH, LGALS3, AKT2, CHEK2, CHEK1, NAMPT, TRIM24, KDR, AKT1, LGALS9, LGALS7, GLUL, CSNK2A1, DAPK1, PRKCB, PRKCA, AR, BTK, TP53, CFTR, PRKDC, EPAS1, PIK3R1, HIF1A, NUAK1, TERT, HMOX1, LYN, PLK4, PLK3, JUN, CREBBP, PLK1, PTK6, BRAF, PTK2, NET1, NOX4, APOBEC3A, TOP2A, ACHE, ITK, FEN1, KIF11, GLI1, GLI2, IKBKB, XPO1, HNF4A, RAC1, JAK2, IKBKE, JAK1, PARP1, SYK, PRMT1, FOS, SIRT1, SIRT2, SIRT3, MMP12, HCK, MMP14, INCENP, RARA, RARB, PPARG, MAPT, PABPC1, SGK1, PPARA, PPARD, PCNA, NR1I3, GSTP1, XIAP, TYMS, RELA, EGFR, PPP2CA, PTPA, RXRA, STAT6, BRD4, STAT1, MGMT, NR1H2, STAT3, MBD2, AKR1C3, NR1H3, PPP1CA, FABP1, FABP3, FABP5, APEX1, HSPA1A, KDM1A, RORC, RORA, AHR, NR3C1, FGF2, MPO, IGF1R, CCND2, CCND1, TUBB3, PIM1, EP300, NEK2, TGM2, PDGFRB, PDGFRA, CASR, MAP2K1, IGFBP3, ADAM10, CCNE1, RAF1, PPIA, VCP, CBFB, TNKS, PSEN2, PSEN1, ADRB2, HSP90B1, CCNB2, CCNB1, ABL1, MAP3K20, NLRP3, MCL1, HSPA8, HSPA5, VDR, ESR1, ESR2, CLK2, CDK9, CDK7, CDK6, CDK5, CDK4, CDK2, BCL2, MDM2, CDK1, MDM4, HPSE, FGFR3, FGFR1, GSK3B, HSP90AB1, THRB, THRA, FLT3, ITGB3, PIK3CB, ADRA1B, ADRA1A, POLB, CASP9, CASP7, CASP3, NOS1, HSP90AA1, PDPK1, PDE4D, MMP2, MMP3, DNMT3A, TGFBR1, CCNA2, BIRC5, MET, HDAC4, HDAC5, HDAC2, HDAC3, ROCK2, HDAC1, TTK, HDAC8, HDAC9, HDAC6, AURKB, AURKA, HDAC7, MAPK9, MAPK8, ERBB4, ERBB2, MAPK1, MAPK3, NOS2, NOS3, LIMK1, PTPN11, MAPK14, MTOR, WEE1, ACKR3, ERCC5, PTPN6 |
| Cellular Component | GO:0005654 | nucleoplasm | 171 | 4.82E-24 | KDM1A, CCNH, RORC, RORA, AHR, NR3C1, MPO, NR3C2, LGALS3, HPGDS, CCND2, TBK1, CCND1, CHEK2, AKT2, CHEK1, TRIM24, PIM1, AKT1, EP300, NEK2, EPHB2, PDGFRA, CSNK2A1, PRKCB, PRKCA, MIF, AR, CCNE1, PGR, TP53, EPHA3, ABCG2, CSF1R, VCP, PFKFB3, ABCB1, CBFB, TNKS, PRKDC, EPAS1, GLO1, PSEN1, HIF1A, HSD11B2, CCNB1, NUAK1, TERT, ABL1, HMOX1, MCL1, PLK3, HSPA8, CREBBP, JUN, VDR, PLK1, PTK6, BMX, ESR1, ESR2, CLK2, CDK9, CDK7, CDK6, CDK5, CDK4, CDK2, BCL2, MDM2, CDK1, UBA2, NOX4, MDM4, HPSE, APOBEC3A, TOP2A, GSK3B, FEN1, THRB, HSP90AB1, THRA, FLT4, ITGB3, AKR1B1, PIK3CB, GLI1, ADRA1A, GLI2, POLB, CASP7, CASP8, XPO1, CASP3, HNF4A, CTSK, NOS1, JAK2, IKBKE, HRAS, HSP90AA1, PARP1, PRMT1, DNMT3A, FOS, SIRT1, SIRT3, CCNA2, INCENP, RARA, RARB, BIRC5, PPARG, SGK1, PPARA, TKT, MET, PPARD, HDAC4, HDAC5, HDAC2, HDAC3, PCNA, ADH1C, SRC, HDAC1, ADH1A, NR1I3, NR1I2, XIAP, HDAC8, HDAC9, HDAC6, RELA, AURKB, HDAC7, AURKA, PTPA, MAPK9, PAK1, MAPK8, RXRA, ERBB4, ALOX5, CXCR2, S1PR1, MAPK1, STAT6, BRD4, MAPK3, NQO2, NOS2, STAT1, MGMT, NR1H2, STAT3, MBD2, NR1H3, PTPN11, MAPK14, MTOR, PPP1CA, FABP1, WEE1, FABP5, APEX1, ERCC5, PTPN6, HSPA1A |
| Cellular Component | GO:0016021 | integral component of membrane | 137 | 0.003423325 | CHRM2, APP, CD81, COMT, ATP12A, TNF, ICAM1, IGF1R, EDNRB, STS, CYP2D6, ANPEP, SCN9A, ADORA1, EPHB2, EPHB1, PDGFRB, CHRNB2, KCNH2, EPHA5, CASR, ST6GAL1, CPT1A, F2R, ADAM10, MPEG1, AR, SMO, SOAT1, AGTR1, IL6ST, TP53, CFTR, EPHA3, ABCG2, CSF1R, CHRNA3, ABCB1, CHRNA5, CHRNA4, PSEN2, FPR1, LPAR1, LPAR2, PSEN1, FPR2, MST1R, ADRB2, CYP19A1, FUT4, DPP4, UGCG, HSD11B2, EBP, HRH2, HMOX1, COX2, COX1, DRD2, MCL1, ST3GAL3, ACE, INSR, EPHX1, SELE, ESR1, GRIN2B, ATP4A, PTPRC, BCL2, NOX4, FGFR3, NOX1, BCL2L1, FGFR1, RET, ALK, ACHE, GSK3B, FLT3, ECE1, ADRA1D, GRIK2, PCSK7, ADRA1B, ADRA1A, ADAMTS4, CTSK, BDKRB2, CA9, ACP1, CCR4, ABCC1, MME, GPR35, OPRM1, TACR1, F3, TGFBR1, TGFBR2, MMP14, MMP13, CCKBR, ADORA2A, KCNMA1, KIT, TLR9, MET, PTGER4, OPRD1, HDAC2, PTGER3, HMGCR, HSD17B7, CYP3A4, EGFR, CXCR1, ERBB4, CXCR3, ERBB2, HSD17B2, CXCR2, S1PR1, KISS1R, BCHE, PTPN1, SLC16A1, MERTK, HCRTR1, P2RX7, AXL, F2RL1, ACKR3, ERCC5 |
| Cellular Component | GO:0016020 | membrane | 126 | 5.51E-09 | CHRM2, APP, EIF4A1, CD81, COMT, NR3C1, TNF, ICAM1, IGF1R, LGALS3, CCND2, STS, CCND1, CAPN1, PDGFRB, CHRNB2, PDGFRA, CPT1A, PRKCH, ADAM10, PRKCA, MELK, SMO, SOAT1, AGTR1, EZR, IL6ST, PPIA, CFTR, CHRNA3, ABCB1, CBFB, CHRNA4, PRKDC, ALOX15, PSEN2, FPR1, ALOX12, PSEN1, FPR2, ADRB2, PIK3R1, CYP19A1, PRKCZ, FUT4, HSP90B1, DPP4, CCNB2, UGCG, CCNB1, GRK2, HMOX1, NLRP3, COX2, MCL1, HSPA8, ACE, HSPA5, INSR, LNPEP, ESR1, CDK9, PTPRC, CDK5, FASN, BCL2, CDK1, NOX4, REN, HPSE, NOX1, FGFR1, RET, ACHE, FEN1, HSP90AB1, PIK3CD, ECE1, PIK3CB, PCSK7, KIF11, PIK3CG, GLI2, MMP25, XPO1, CA9, RAC1, JAK2, GUSB, JAK3, HRAS, JAK1, ABCC1, HSP90AA1, PARP1, MME, PDPK1, PDE4D, FOS, F3, TGFBR1, TGFBR2, ADORA2A, PIK3CA, KCNMA1, MAPT, PABPC1, MET, PTGER4, PTGER3, TTK, EGFR, PPP2CA, CDC42, ERBB2, CXCR2, KISS1R, SLC16A1, MGMT, LIMK1, MBD2, MTOR, VEGFA, P2RX7, PTPN6 |
| Cellular Component | GO:0070062 | extracellular exosome | 92 | 7.14E-11 | ALK, EIF4A1, APP, HSP90AB1, CD81, ITGB3, SERPINE1, AKR1B1, PLAT, ECE1, COMT, MPO, ICAM1, LGALS3, ALDH2, TUBB3, PLAU, ANPEP, NAMPT, CTSF, RAC1, CAPN1, GUSB, LGALS7, ACP1, GLUL, CTSD, EPHB1, EPHB4, TGM2, CTSB, CBR1, ABCC1, HSP90AA1, PRKCH, MMP7, MME, PRKCB, AKR1A1, ADAM10, PRKCA, MIF, F2, MMP9, SMO, PABPC1, EZR, TKT, IL6ST, PPIA, VCP, PCNA, ABCB1, SRC, GSTP1, GLO1, PON1, PLG, ALOX12, PRKCZ, HSP90B1, PTGS1, PPP2CA, DPP4, PTPA, CDC42, TTR, CSK, LYN, HSPA8, GSTM2, NQO2, SLC16A1, ACE, YES1, HSPA5, INSR, IDH1, PLA2G2A, AKR1C3, PPP1CA, FABP1, FABP3, PTPRC, FABP5, AXL, FASN, CDK1, PTPN6, SHBG, HSPA1A |
| Cellular Component | GO:0005887 | integral component of plasma membrane | 91 | 4.61E-22 | RET, CHRM2, ALK, APP, CHRM3, FLT1, FLT3, CD81, PTGDR2, FLT4, ITGB3, ADRA1D, GRIK2, ADRA1B, ADRA1A, TNF, IGF1R, ICAM1, EDNRA, EDNRB, SCN9A, ADORA1, KDR, BDKRB2, EPHB2, CCR4, EPHB1, EPHB4, EPHB3, PDGFRB, KCNH2, CHRNB2, EPHA5, PDGFRA, ABCC1, CASR, MME, GPR35, EPHA8, F2R, TACR1, OPRM1, ADRA2B, F3, TGFBR2, MMP14, ADORA2A, KIT, AGTR1, EPHA1, MET, CFTR, EPHA3, EPHA2, OPRD1, CSF1R, CHRNA3, CHRNA5, CHRNA4, C5AR1, PTGER3, PSEN2, LPAR1, LPAR2, MST1R, ADRB2, PSEN1, EGFR, ERBB4, HRH2, CXCR3, ERBB2, CXCR2, KISS1R, DRD2, NTRK1, SLC16A1, INSR, LNPEP, MERTK, SELE, GRIN2B, HCRTR1, P2RX7, ATP4A, PTPRC, AXL, F2RL1, TEK, FGFR3, FGFR1 |
| Cellular Component | GO:0005576 | extracellular region | 83 | 1.18E-08 | ACHE, APP, HSP90AB1, FLT4, SERPINE1, PLAT, MPO, FGF2, TNF, ADAMTS4, ADAMTS5, LGALS3, MMP25, SHH, PLAU, CTSK, KDR, CAPN1, EPHB2, GUSB, CTSD, EPHB1, EPHB4, EPHB3, CTSB, ST6GAL1, HSP90AA1, MMP7, MMP1, IGFBP3, MMP2, MMP3, F2R, MIF, F2, MMP9, MPEG1, MMP10, TGFBR2, MMP12, MMP13, TLR9, MAPT, IL6ST, MET, PPIA, EPHA3, VCP, ROCK1, GSTP1, PON1, PLG, HSP90B1, DPP4, TTR, ERBB4, ALOX5, MAPK1, NLRP3, ST3GAL3, BCHE, HSPA8, ACE, CMA1, IDH1, PLA2G2A, LNPEP, MAPK14, IL2, VEGFA, IL6, BMP1, AKR1B10, FABP5, PTPN6, REN, TEK, HPSE, SHBG, ALPG, FGFR3, FGFR1, HSPA1A |
| Cellular Component | GO:0005615 | extracellular space | 69 | 9.55E-06 | ACHE, APP, FLT1, SERPINE1, AKR1B1, PLAT, MPO, FGF2, TNF, ICAM1, ADAMTS4, ADAMTS5, LGALS3, SHH, CASP7, PLAU, ANPEP, CTSK, NAMPT, CHEK1, CASP1, CTSF, LGALS9, GUSB, LGALS7, CTSD, CTSB, MMP7, IGFBP3, MMP2, MMP3, AKR1A1, MIF, F2, MMP9, F3, MMP10, MMP12, MMP14, MMP13, KIT, EZR, IL6ST, PPIA, GSTP1, PON1, PLG, EGFR, TTR, ALOX5, HMOX1, XDH, BCHE, HSPA8, ACE, CMA1, PLA2G2A, MERTK, SELE, IL2, VEGFA, ATP4A, IL6, FABP3, BMP1, FABP5, AXL, REN, HPSE |
| Cellular Component | GO:0032991 | macromolecular complex | 66 | 1.89E-25 | ALK, TOP2A, APP, FEN1, HSP90AB1, KDM1A, ITGB3, AHR, KIF11, NR3C1, TNF, POLB, CASP9, CASP8, XPO1, AKT2, CHEK1, CASP1, AKT1, NEK2, NOS1, GLUL, PDGFRA, HSP90AA1, PARP1, SYK, RIPK2, SIRT1, SIRT3, AR, INCENP, RARA, BIRC5, EZR, PPIA, TP53, CFTR, VCP, HDAC2, PRKDC, HDAC1, PSEN2, PSEN1, PIK3R1, PTGS2, HIF1A, HDAC6, EGFR, HSP90B1, CDC42, PAK1, TTR, ABL1, NTRK1, PTPN1, HSPA5, STAT1, MBD2, PTPN11, ESR1, FABP1, BCL2, MDM2, PTPN6, ERCC5, HSPA1A |
| Cellular Component | GO:0005739 | mitochondrion | 58 | 1.42E-06 | APP, GSK3B, FEN1, HSP90AB1, NR3C1, CASP9, CASP8, ALDH2, CYP2D6, TRIM24, AKT1, CAPN1, NOS1, GLUL, TGM2, PDK1, CPT1A, MAP2K1, HSP90AA1, PARP1, MMP2, PRKCA, SIRT1, SIRT2, SIRT3, MAPT, RAF1, SGK1, TP53, SRC, GSTP1, PSEN1, TYMS, PPP2CA, MAPK9, MAPK8, RXRA, ERBB4, ABL1, COX2, MAPK1, NLRP3, COX1, MCL1, MAPK3, HSPA5, IDH1, BRAF, MAPK14, ESR2, P2RX7, AKR1B10, APEX1, BCL2, CDK1, NOX4, BCL2L1, HSPA1A |
| Cellular Component | GO:0009986 | cell surface | 49 | 7.48E-15 | ACHE, APP, HSP90AB1, ITGB3, PLAT, TNF, ICAM1, LGALS3, SHH, PLAU, EPHB2, PDGFRB, KCNH2, CASR, HSP90AA1, MME, F2R, ADAM10, MIF, TACR1, ADRA2B, F3, TGFBR1, RARA, MET, CFTR, EPHA2, CSF1R, ABCB1, PSEN2, LPAR1, LPAR2, PLG, MST1R, PSEN1, EGFR, FUT4, DPP4, CXCR2, KISS1R, NTRK1, HSPA5, GRIN2B, VEGFA, PTPRC, AXL, ACKR3, TEK, FGFR3 |
| Cellular Component | GO:0043231 | intracellular membrane-bounded organelle | 49 | 1.56E-08 | PIK3CB, COMT, MPO, ADRA1A, IGF1R, POLB, HPGDS, STS, TBK1, XPO1, CYP2D6, AKT2, CTSK, CHEK1, BDKRB2, CTSF, GUSB, PDGFRB, ADAM10, ADRA2B, HCK, SMO, PPARG, PPIA, CSF1R, VCP, PON1, CYP2C19, CYP3A4, PTGS2, PTGS1, HSD11B2, MAPK9, MAPK8, HSD17B2, KISS1R, S1PR1, LYN, SLC16A1, HSPA5, CMA1, BRAF, ESR2, PTK2, AXL, CYP1A2, CYP1A1, ACKR3, HPSE |
| Cellular Component | GO:0048471 | perinuclear region of cytoplasm | 46 | 6.85E-11 | APP, ACHE, VCP, HSP90AB1, SRC, PSEN2, ECE1, PSEN1, PIK3R1, PRKCZ, HDAC6, EGFR, AURKA, HSP90B1, ALOX5, ERBB2, ABL1, HMOX1, NOS1, HRAS, TGM2, CTSB, LYN, KCNH2, EPHA5, HSPA8, HSP90AA1, NOS2, STAT1, PDE4D, PLA2G2A, PRKCA, LNPEP, SELE, SIRT2, PTK2, CDK7, PIK3CA, APEX1, RARA, BTK, NOX4, ACKR3, PPARG, EZR, HSPA1A |
| Cellular Component | GO:0000785 | chromatin | 46 | 2.95E-06 | HDAC2, THRB, PCNA, THRA, KDM1A, EPAS1, HDAC1, NR1I3, NR1I2, RORC, RORA, AHR, NR3C1, HIF1A, RELA, NR3C2, RXRA, CCND2, HNF4A, CHEK1, STAT6, TGM2, BRD4, JUN, CREBBP, PARP1, STAT1, VDR, NR1H2, PLK1, MBD2, STAT3, NR1H3, FOS, ESR1, SIRT1, ESR2, AR, CDK4, RARA, RARB, PPARG, PGR, PPARA, TP53, PPARD |
| Cellular Component | GO:0043235 | receptor complex | 43 | 1.31E-28 | RET, ALK, CSF1R, APP, FLT1, FLT3, FLT4, ITGB3, MST1R, ADRB2, EGFR, NR3C2, IGF1R, RXRA, ERBB4, ERBB2, KDR, EPHB2, EPHB1, EPHB4, EPHB3, PDGFRB, NTRK1, EPHA5, PDGFRA, VDR, EPHA8, INSR, NR1H3, MERTK, TGFBR1, TGFBR2, AXL, KIT, PPARG, TEK, IL6ST, EPHA1, MET, FGFR3, EPHA3, FGFR1, EPHA2 |
| Cellular Component | GO:0005789 | endoplasmic reticulum membrane | 43 | 1.02E-04 | CHRM3, VCP, PON1, PSEN2, HMGCR, PSEN1, CYP2C19, HSD17B7, CYP3A4, PTGS2, CYP19A1, EGFR, HSP90B1, NR3C2, PTGS1, CDC42, HSD11B2, SHH, EBP, STS, CYP2D6, HSD17B2, HMOX1, RAC1, HRAS, PDGFRA, HSPA5, EPHX1, PLA2G2A, GRIN2B, MTOR, SOAT1, CYP1A2, CYP1A1, BCL2, CDK1, TLR9, NOX4, SGK1, TKT, CFTR |
| Cellular Component | GO:0005925 | focal adhesion | 41 | 1.13E-15 | FLT1, CD81, SRC, ITGB3, EGFR, HSP90B1, ICAM1, DPP4, CDC42, PAK1, PLAU, MAPK1, RAC1, CAPN1, JAK2, JAK1, TGM2, MAPK3, MAP4K4, PDGFRB, HSPA8, MAP2K1, YES1, MME, HSPA5, PDPK1, LIMK1, ADAM10, OPRM1, PTK2, HCK, MMP14, PTPRC, NOX4, TEK, PABPC1, EZR, FGFR3, PPIA, HSPA1A, EPHA2 |
| Molecular Function | GO:0005515 | protein binding | 372 | 2.42E-24 | PNMT, APP, EIF4A1, CCNH, SERPINE1, MYLK, ICAM1, LGALS3, EDNRA, EDNRB, TBK1, AKT2, CHEK2, CHEK1, NAMPT, TRIM24, KDR, AKT1, EPHB2, LGALS7, GLUL, EPHB1, EPHB4, EPHB3, PDK1, EPHA5, ST6GAL1, PRKCH, CSNK2A1, DAPK1, PRKCB, PRKCA, MIF, AR, SOAT1, BTK, AGTR1, EZR, TP53, CFTR, EPHA3, EPHA2, ABCB1, PRKDC, EPAS1, FPR1, LPAR1, LPAR2, PIK3R1, FPR2, MST1R, HIF1A, PRKCZ, UGCG, NUAK1, TERT, HMOX1, COX2, COX1, ST3GAL3, LYN, PLK4, PLK3, JUN, CREBBP, GSTM2, YES1, INSR, PLK1, PTK6, BRAF, LNPEP, GRIN2B, PTK2, NET1, ATP4B, AKR1B10, FASN, CYP1A2, CYP1A1, NOX4, REN, APOBEC3A, NOX1, RET, TOP2A, ACHE, ITK, FEN1, AKR1B1, KIF11, PCSK7, GLI1, GLI2, IKBKB, SHH, MMP25, XPO1, HNF4A, CTSK, RAC1, JAK2, JAK3, HRAS, CCR4, IKBKE, CTSD, JAK1, CTSB, PARP1, SYK, MME, RIPK2, PRMT1, AKR1A1, TACR1, FOS, F2, F3, SIRT1, SIRT2, SIRT3, HCK, MMP14, CCKBR, INCENP, KCNMA1, KIT, RARA, PPARG, MAPT, PABPC1, SGK1, PPARA, TKT, PPARD, PTGER4, OPRD1, PCNA, ADH1A, NR1I3, GSTP1, NR1I2, ODC1, XIAP, PTGS2, RELA, EGFR, PTGS1, TYMP, PPP2CA, PTPA, PAK1, RXRA, CXCR1, HSD17B1, ALOX5, CXCR3, CXCR2, KISS1R, STAT6, XDH, MAP4K4, BRD4, BCHE, NQO2, SLC16A1, STAT1, NR1H2, STAT3, MBD2, NR1H3, VEGFA, PPP1CA, FABP1, FABP3, FABP5, AXL, APEX1, F2RL1, HSPA1A, CHRM3, KDM1A, CD81, RORC, PLAT, RORA, AHR, COMT, NR3C1, FGF2, MPO, TNF, NR3C2, IGF1R, HPGDS, CCND2, CCND1, TUBB3, PLAU, ADORA1, PIM1, EP300, NEK2, MAP3K8, PIM3, PIM2, CAPN1, TGM2, PDGFRB, CHRNB2, KCNH2, PDGFRA, CASR, MAP2K1, IGFBP3, F2R, ADAM10, MELK, SMO, CCNE1, PGR, RAF1, IL6ST, PPIA, ABCG2, CSF1R, CHRNA3, VCP, PFKFB3, CHRNA5, CBFB, CHRNA4, TNKS, GLO1, ALOX15, PSEN2, ALOX12, PSEN1, ADRB2, HSP90B1, DPP4, CCNB2, CCNB1, EBP, GRK2, ABL1, MAP3K20, NLRP3, DRD2, MCL1, HSPA8, HSPA5, VDR, IDH1, EPHX1, BMX, ESR1, SELE, ESR2, IL2, CLK2, CDK9, IL6, CDK7, BMP1, PTPRC, CDK6, CDK5, CDK4, CDK2, BCL2, MDM2, CDK1, UBA2, MDM4, HPSE, SHBG, FGFR3, BCL2L1, FGFR1, ALK, GSK3B, HSP90AB1, FLT1, THRB, THRA, FLT3, ITGB3, FLT4, PIK3CD, ADRA1D, ECE1, PIK3CB, ADRA1B, ADRA1A, PIK3CG, ADAMTS4, POLB, CASP9, ADAMTS5, CASP7, CASP8, CASP3, BDKRB2, CASP1, CA9, NOS1, ACP1, HSP90AA1, MMP7, PDPK1, PDE4D, MMP2, MMP3, DNMT3A, OPRM1, ADRA2B, MMP9, TGFBR1, TGFBR2, CCNA2, ADORA2A, PIK3CA, BIRC5, MET, HDAC4, HDAC5, HDAC2, HDAC3, BCL2A1, ROCK1, SRC, ROCK2, HDAC1, TTK, PLG, HMGCR, CYP3A4, HDAC8, HDAC9, HDAC6, AURKB, AURKA, HDAC7, CDC42, MAPK9, MAPK8, TTR, ERBB4, ERBB2, S1PR1, MAPK1, CSK, MAPK3, NTRK1, PTPN1, NOS2, NOS3, LIMK1, PTPN11, MERTK, MAPK14, MTOR, P2RX7, WEE1, ACKR3, ERCC5, PTPN6, TEK |
| Molecular Function | GO:0005524 | ATP binding | 135 | 9.52E-46 | EIF4A1, ATP12A, MYLK, IGF1R, TBK1, CHEK2, AKT2, CHEK1, PIM1, KDR, AKT1, MAP3K8, PIM3, NEK2, PIM2, EPHB2, GLUL, EPHB1, EPHB4, EPHB3, PDK1, TGM2, PDGFRB, EPHA5, PDGFRA, MAP2K1, PRKCH, CSNK2A1, DAPK1, PRKCB, EPHA8, PRKCA, MELK, BTK, RAF1, EPHA1, CFTR, EPHA3, EPHA2, ABCG2, CSF1R, VCP, PFKFB3, ABCB1, PRKDC, MST1R, PRKCZ, HSP90B1, NUAK1, GRK2, ABL1, MAP3K20, NLRP3, LYN, PLK4, PLK3, HSPA8, YES1, HSPA5, INSR, PLK1, PTK6, BRAF, BMX, PTK2, CLK2, CDK9, ATP4A, CDK7, CDK6, CDK5, CDK4, CDK2, CDK1, UBA2, FGFR3, FGFR1, RET, ALK, TOP2A, GSK3B, ITK, FLT1, HSP90AB1, FLT3, FLT4, PIK3CD, PIK3CB, KIF11, PIK3CG, IKBKB, JAK2, JAK3, IKBKE, JAK1, ABCC1, HSP90AA1, SYK, PDPK1, RIPK2, TGFBR1, TGFBR2, HCK, PIK3CA, KIT, SGK1, MET, ROCK1, ROCK2, SRC, TTK, EGFR, AURKB, AURKA, PTPA, MAPK9, PAK1, MAPK8, ERBB4, ERBB2, MAPK1, CSK, MAPK3, MAP4K4, NTRK1, LIMK1, MAPK14, MERTK, MTOR, P2RX7, WEE1, AXL, TEK, HSPA1A |
| Molecular Function | GO:0004712 | protein serine/threonine/tyrosine kinase activity | 102 | 4.96E-74 | IGF1R, TBK1, CHEK2, AKT2, CHEK1, PIM1, KDR, AKT1, MAP3K8, PIM3, NEK2, PIM2, EPHB2, EPHB1, EPHB4, EPHB3, PDGFRB, EPHA5, PDGFRA, MAP2K1, PRKCH, CSNK2A1, DAPK1, PRKCB, EPHA8, PRKCA, MELK, BTK, RAF1, EPHA1, EPHA3, EPHA2, CSF1R, PRKDC, MST1R, PRKCZ, NUAK1, ABL1, MAP3K20, LYN, PLK4, PLK3, YES1, INSR, PLK1, PTK6, BMX, PTK2, CLK2, CDK9, CDK7, CDK6, CDK5, CDK4, CDK2, CDK1, FGFR3, FGFR1, RET, ALK, GSK3B, ITK, FLT1, FLT3, FLT4, PIK3CG, IKBKB, JAK2, JAK3, JAK1, SYK, PDPK1, RIPK2, HCK, PIK3CA, KIT, SGK1, MET, ROCK1, ROCK2, SRC, TTK, EGFR, AURKB, AURKA, MAPK9, PAK1, MAPK8, ERBB4, ERBB2, MAPK1, CSK, MAPK3, MAP4K4, NTRK1, LIMK1, MAPK14, MERTK, MTOR, WEE1, AXL, TEK |
| Molecular Function | GO:0042802 | identical protein binding | 101 | 4.23E-20 | APP, NR3C1, FGF2, TNF, IGF1R, TBK1, CHEK2, NAMPT, KDR, AKT1, EPHB2, GLUL, KCNH2, CPT1A, CSNK2A1, DAPK1, MIF, SOAT1, BTK, PGR, EZR, RAF1, IL6ST, TP53, ABCG2, VCP, ADRB2, DPP4, EBP, TERT, HMOX1, NLRP3, DRD2, PLK4, JUN, INSR, IDH1, PLK1, PTK6, BRAF, ESR1, CLK2, BMP1, BCL2, MDM2, FGFR3, BCL2L1, FGFR1, ALK, HSP90AB1, ITGB3, ADRA1D, GRIK2, PIK3CG, CASP9, IKBKB, ADAMTS5, CASP8, CASP1, JAK2, IKBKE, HSP90AA1, PARP1, RIPK2, PRMT1, DNMT3A, FOS, SIRT1, MMP9, ADORA2A, KCNMA1, BIRC5, PPARG, MAPT, MET, HDAC4, HDAC5, PCNA, XIAP, TTK, RELA, EGFR, CDC42, PAK1, TTR, RXRA, ERBB2, MAPK1, STAT6, CSK, MAPK3, NTRK1, BCHE, SLC16A1, STAT1, STAT3, MTOR, VEGFA, P2RX7, FABP5, TEK |
| Molecular Function | GO:0046872 | metal ion binding | 80 | 0.005156464 | TOP2A, ITK, THRB, ECE1, GLI1, ATP12A, MPO, MYLK, GLI2, ADAMTS4, POLB, SHH, STS, TUBB3, CHEK2, AKT2, EP300, NEK2, NOS1, JAK2, JAK1, TGM2, PRKCH, MME, PDE4D, IGFBP3, DNMT3A, ADAM10, SIRT1, TGFBR1, TGFBR2, KCNMA1, KIT, BTK, BIRC5, PPARG, RAF1, TKT, TP53, IDO1, HDAC4, HDAC5, ROCK1, ROCK2, HDAC1, NR1I3, PON1, XIAP, HDAC8, PTGS2, HDAC9, PRKCZ, AURKB, HDAC7, PTGS1, PPP2CA, NUAK1, TERT, COX2, HMOX1, COX1, CSK, CREBBP, ACE, NOS2, NOS3, IDH1, LIMK1, MBD2, BMX, SELE, ESR1, PPP1CA, ATP4A, APEX1, MDM2, ERCC5, MDM4, ALPG, NOX1 |
| Molecular Function | GO:0004672 | protein kinase activity | 67 | 7.49E-40 | RET, GSK3B, FLT4, PIK3CG, MYLK, IKBKB, TBK1, CCND1, CHEK2, AKT2, CHEK1, TRIM24, AKT1, PIM3, NEK2, MAP3K8, PIM2, JAK2, IKBKE, PDK1, PDGFRA, MAP2K1, PRKCH, CSNK2A1, SYK, PDPK1, RIPK2, PRKCB, DAPK1, PRKCA, TGFBR1, MELK, RAF1, EPHA1, MET, ROCK1, ROCK2, SRC, PRKDC, PRKCZ, EGFR, AURKA, MAPK9, NUAK1, PAK1, GRK2, ERBB2, ABL1, MAP3K20, MAP4K4, NTRK1, LIMK1, PLK1, BRAF, MAPK14, MTOR, PTK2, CLK2, CDK9, WEE1, CDK7, CDK5, CDK4, CDK2, CDK1, TEK |
| Molecular Function | GO:0019899 | enzyme binding | 66 | 1.13E-38 | TOP2A, APP, THRB, HSP90AB1, KDM1A, ITGB3, POLB, CCND1, AKT1, RAC1, LGALS9, PDGFRB, HSP90AA1, PRKCH, PARP1, PRMT1, PDE4D, PRKCA, SIRT1, AR, ADORA2A, RARA, BIRC5, PPARG, PGR, MAPT, RAF1, TP53, CFTR, HDAC2, HDAC3, PCNA, SRC, PRKDC, HDAC1, PLG, MST1R, CYP2C19, CYP3A4, PTGS2, HIF1A, HDAC6, EGFR, RELA, HSP90B1, MAPK8, RXRA, HMOX1, BRD4, LYN, PTPN1, BCHE, HSPA8, GSTM2, JUN, YES1, HSPA5, STAT1, MAPK14, ESR1, ESR2, CYP1A2, CYP1A1, MDM2, MDM4, HSPA1A |
| Molecular Function | GO:0008270 | zinc ion binding | 65 | 1.99E-17 | THRB, THRA, RORC, RORA, ECE1, NR3C1, GLI2, NR3C2, ADAMTS5, MMP25, SHH, ANPEP, HNF4A, TRIM24, EP300, CA9, MMP7, PARP1, MME, PRKCB, MMP1, MMP2, MMP3, PRKCA, MMP9, SIRT2, MMP10, SIRT3, MMP12, AR, MMP14, MMP13, RARA, RARB, BIRC5, PPARG, PGR, PPARA, TP53, PPARD, HDAC4, ADH1C, TNKS, GLO1, NR1I3, ADH1A, NR1I2, HDAC6, RXRA, PTPN1, NQO2, CREBBP, ACE, VDR, NR1H2, NR1H3, LNPEP, ESR1, GRIN2B, ESR2, BMP1, MDM2, MDM4, APOBEC3A |
| Molecular Function | GO:0004674 | protein serine/threonine kinase activity | 58 | 9.08E-30 | GSK3B, PIK3CG, IKBKB, TBK1, CHEK2, AKT2, CHEK1, PIM1, AKT1, PIM3, NEK2, MAP3K8, PIM2, IKBKE, MAP2K1, PRKCH, CSNK2A1, SYK, PDPK1, RIPK2, PRKCB, DAPK1, PRKCA, TGFBR1, TGFBR2, MELK, PIK3CA, RAF1, SGK1, ROCK1, ROCK2, PRKDC, TTK, PRKCZ, AURKB, AURKA, MAPK9, NUAK1, PAK1, MAPK8, MAP3K20, MAPK1, MAPK3, MAP4K4, PLK4, PLK3, LIMK1, PLK1, BRAF, MAPK14, MTOR, CLK2, CDK9, CDK7, CDK5, CDK4, CDK2, CDK1 |
| Molecular Function | GO:0042803 | protein homodimerization activity | 55 | 7.60E-15 | TOP2A, ACHE, APP, HSP90AB1, FLT4, ECE1, AHR, IKBKB, HPGDS, CHEK2, HNF4A, AKT1, KCNH2, PDGFRA, CASR, ST6GAL1, HSP90AA1, PARP1, MME, RIPK2, ADAM10, KIT, TLR9, BIRC5, TKT, ABCG2, CSF1R, BCL2A1, PON1, ODC1, ADRB2, TYMS, PTGS2, RELA, TYMP, DPP4, PTPA, TERT, ERBB4, HSD17B1, HMOX1, XDH, MCL1, NTRK1, GSTM2, NQO2, NOS2, STAT1, IDH1, STAT3, VEGFA, BCL2, ERCC5, FGFR1, BCL2L1 |
| Molecular Function | GO:0003677 | DNA binding | 46 | 0.003233921 | HDAC4, TOP2A, APP, FEN1, THRB, PCNA, EPAS1, NR1I3, RORC, RORA, AHR, PIK3R1, NR3C1, GLI1, HIF1A, RELA, POLB, TERT, HNF4A, ABL1, MAPK1, EP300, STAT6, JUN, PARP1, MGMT, STAT1, VDR, NR1H2, MBD2, STAT3, DNMT3A, NR1H3, ESR1, ESR2, CDK9, AR, APEX1, RARB, PPARG, PGR, MAPT, PPARA, TP53, PPARD |
| Molecular Function | GO:0004713 | protein tyrosine kinase activity | 44 | 5.23E-42 | RET, ALK, CSF1R, ITK, FLT1, FLT3, SRC, FLT4, TTK, MST1R, EGFR, IGF1R, ERBB4, ERBB2, KDR, ABL1, CSK, EPHB2, JAK2, JAK3, JAK1, LYN, PDGFRB, NTRK1, MAP2K1, YES1, SYK, INSR, BRAF, PTK6, BMX, MERTK, PTK2, CLK2, HCK, WEE1, AXL, KIT, BTK, TEK, MET, FGFR3, FGFR1, EPHA2 |
| Molecular Function | GO:0019901 | protein kinase binding | 42 | 1.90E-12 | HDAC4, GSK3B, HSP90AB1, KIF11, NR3C1, HIF1A, PRKCZ, RELA, AURKA, HDAC7, CASP9, IKBKB, CDC42, CCNB1, CCND2, CCND1, CHEK2, ABL1, AKT1, RAC1, JAK2, PDGFRB, PTPN1, CASR, SYK, PARP1, PLK1, STAT3, ADAM10, PTPN11, ESR1, PTK2, CDK9, CCNA2, PTPRC, CDK5, CCNE1, PTPN6, MAPT, EPHA1, TP53, BCL2L1 |
| Molecular Function | GO:0000978 | RNA polymerase II core promoter proximal region sequence-specific DNA binding | 41 | 0.009159454 | HDAC4, HDAC5, APP, THRB, THRA, EPAS1, HDAC1, NR1I3, NR1I2, RORC, RORA, NR3C1, GLI1, HIF1A, RELA, HDAC6, NR3C2, GLI2, RXRA, HNF4A, STAT6, JUN, STAT1, VDR, NR1H2, STAT3, DNMT3A, NR1H3, FOS, ESR1, SIRT1, ESR2, CDK9, AR, RARA, RARB, PPARG, PGR, PPARA, TP53, PPARD |
| Molecular Function | GO:0043565 | sequence-specific DNA binding | 34 | 1.70E-12 | HDAC4, HDAC2, THRB, THRA, CBFB, EPAS1, NR1I3, NR1I2, RORC, RORA, NR3C1, GLI1, HIF1A, NR3C2, GLI2, RXRA, HNF4A, NLRP3, VDR, NR1H2, NR1H3, FOS, ESR1, ESR2, SIRT3, MMP12, AR, BCL2, PPARG, PGR, MAPT, PPARA, MET, PPARD |
| Molecular Function | GO:0005102 | receptor binding | 34 | 1.95E-10 | APP, SRC, SERPINE1, PLAT, PLG, DPP4, PTPA, HNF4A, TRIM24, EPHB2, JAK2, GUSB, LYN, PDGFRB, GSTM2, YES1, SYK, RIPK2, F2R, STAT3, ADAM10, PTK6, F2, TGFBR1, PTK2, P2RX7, AR, HCK, PTPRC, RARA, F2RL1, REN, PGR, HSPA1A |
| Molecular Function | GO:0004714 | transmembrane receptor protein tyrosine kinase activity | 32 | 6.17E-38 | RET, ALK, CSF1R, FLT1, FLT3, FLT4, MST1R, EGFR, IGF1R, ERBB4, ERBB2, KDR, EPHB2, EPHB1, EPHB4, EPHB3, PDGFRB, NTRK1, EPHA5, PDGFRA, EPHA8, INSR, MERTK, AXL, KIT, TEK, EPHA1, MET, FGFR3, EPHA3, FGFR1, EPHA2 |
| Molecular Function | GO:0003700 | transcription factor activity, sequence-specific DNA binding | 31 | 9.10E-06 | THRB, THRA, EPAS1, NR1I3, NR1I2, RORC, RORA, AHR, NR3C1, HIF1A, RELA, NR3C2, GLI2, RXRA, HNF4A, STAT6, JUN, STAT1, VDR, NR1H2, STAT3, FOS, ESR1, ESR2, AR, RARA, PPARG, PGR, PPARA, TP53, PPARD |
| Molecular Function | GO:0016301 | kinase activity | 30 | 1.79E-14 | RET, ALK, GSK3B, PRKDC, PIK3CD, PIK3CB, PIK3R1, EGFR, PIK3CG, MYLK, MAPK9, PLAU, CHEK2, ERBB2, ABL1, AKT1, JAK2, LYN, CSNK2A1, DAPK1, RIPK2, PRKCA, MTOR, PIK3CA, CDK5, CCNE1, CDK4, BTK, SGK1, EPHA2 |
| Molecular Function | GO:0003682 | chromatin binding | 30 | 7.62E-07 | TOP2A, APP, HDAC2, HDAC3, PCNA, KDM1A, GLI1, MPO, RELA, EGFR, HDAC7, HNF4A, TRIM24, EP300, BRD4, JUN, CREBBP, PRKCB, MBD2, DNMT3A, FOS, ESR1, SIRT2, CDK9, FABP1, AR, CDK1, RARA, PPARG, TP53 |

**Supplementary Table 5 The significantly enrichment potential term of KEGG pathway analysis**

| **Category** | **KEGG ID** | **KEGG Term** | **Count** | **PValue** | **Genes** |
| --- | --- | --- | --- | --- | --- |
| KEGG  PATHWAY | hsa05200 | Pathways in cancer | 116 | 5.39E-49 | FGF2, IGF1R, EDNRA, EDNRB, CCND2, CCND1, AKT2, PIM1, AKT1, EP300, PIM2, PDGFRB, PDGFRA, MAP2K1, DAPK1, PRKCB, F2R, PRKCA, AR, SMO, CCNE1, AGTR1, RAF1, IL6ST, TP53, CSF1R, EPAS1, LPAR1, LPAR2, PIK3R1, HIF1A, HSP90B1, TERT, ABL1, HMOX1, CREBBP, GSTM2, JUN, BRAF, ESR1, ESR2, PTK2, IL2, IL6, CDK6, CDK4, CDK2, BCL2, MDM2, FGFR3, BCL2L1, FGFR1, RET, ALK, GSK3B, HSP90AB1, FLT3, FLT4, PIK3CD, PIK3CB, GLI1, GLI2, CASP9, IKBKB, SHH, CASP7, CASP8, CASP3, BDKRB2, RAC1, JAK2, JAK3, HRAS, JAK1, HSP90AA1, MMP1, MMP2, FOS, F2, MMP9, TGFBR1, TGFBR2, CCNA2, PIK3CA, KIT, RARA, RARB, BIRC5, PPARG, MET, PPARD, PTGER4, HDAC2, ROCK1, ROCK2, HDAC1, GSTP1, PTGER3, XIAP, PTGS2, RELA, EGFR, CDC42, MAPK9, MAPK8, RXRA, ERBB2, MAPK1, STAT6, MAPK3, NTRK1, NOS2, STAT1, STAT3, MTOR, VEGFA |
| KEGG  PATHWAY | hsa04151 | PI3K-Akt signaling pathway | 71 | 1.66E-26 | CHRM2, GSK3B, FLT1, HSP90AB1, FLT3, FLT4, ITGB3, PIK3CD, PIK3CB, FGF2, PIK3CG, IGF1R, CASP9, IKBKB, CCND2, CCND1, AKT2, KDR, AKT1, RAC1, JAK2, JAK3, HRAS, JAK1, PDGFRB, PDGFRA, MAP2K1, HSP90AA1, SYK, PDPK1, F2R, PRKCA, PIK3CA, CCNE1, KIT, RAF1, SGK1, MET, TP53, EPHA2, CSF1R, LPAR1, LPAR2, PIK3R1, EGFR, RELA, HSP90B1, PPP2CA, RXRA, ERBB4, ERBB2, MAPK1, MCL1, MAPK3, NTRK1, NOS3, INSR, MTOR, IL2, PTK2, VEGFA, IL6, CDK6, CDK4, CDK2, BCL2, MDM2, TEK, FGFR3, FGFR1, BCL2L1 |
| KEGG  PATHWAY | hsa05417 | Lipid and atherosclerosis | 56 | 1.14E-26 | GSK3B, HSP90AB1, PIK3CD, PIK3CB, TNF, ICAM1, CASP9, IKBKB, CASP7, TBK1, CASP8, CASP3, AKT2, CASP1, AKT1, RAC1, JAK2, IKBKE, HRAS, HSP90AA1, PDPK1, MMP1, MMP3, PRKCA, FOS, MMP9, PIK3CA, PPARG, TP53, ROCK2, SRC, PIK3R1, RELA, HSP90B1, CDC42, MAPK9, MAPK8, RXRA, MAPK1, NLRP3, MAPK3, LYN, HSPA8, JUN, HSPA5, NOS3, STAT3, MAPK14, SELE, PTK2, IL6, CYP1A1, BCL2, NOX1, BCL2L1, HSPA1A |
| KEGG  PATHWAY | hsa05205 | Proteoglycans in cancer | 52 | 3.73E-24 | ITGB3, PIK3CD, PIK3CB, FGF2, TNF, IGF1R, SHH, CCND1, PLAU, CASP3, AKT2, KDR, AKT1, RAC1, HRAS, MAP2K1, PDPK1, PRKCB, MMP2, PRKCA, MMP9, PIK3CA, SMO, EZR, RAF1, MET, TP53, ROCK1, ROCK2, SRC, PIK3R1, HIF1A, EGFR, CDC42, PAK1, ERBB4, ERBB2, MAPK1, MAPK3, STAT3, PTPN11, BRAF, MAPK14, ESR1, MTOR, PTK2, PPP1CA, VEGFA, MDM2, PTPN6, HPSE, FGFR1 |
| KEGG  PATHWAY | hsa04010 | MAPK signaling pathway | 52 | 7.40E-17 | FLT1, FLT3, FLT4, FGF2, TNF, IGF1R, IKBKB, CASP3, AKT2, KDR, AKT1, MAP3K8, RAC1, HRAS, PDGFRB, PDGFRA, MAP2K1, PRKCB, PRKCA, FOS, TGFBR1, TGFBR2, KIT, MAPT, RAF1, MET, TP53, EPHA2, CSF1R, EGFR, RELA, CDC42, MAPK9, PAK1, MAPK8, ERBB4, ERBB2, MAP3K20, MAPK1, MAPK3, MAP4K4, NTRK1, HSPA8, JUN, INSR, BRAF, MAPK14, VEGFA, TEK, FGFR3, FGFR1, HSPA1A |
| KEGG  PATHWAY | hsa05206 | MicroRNAs in cancer | 50 | 1.80E-14 | ITGB3, PIK3CD, PIK3CB, IKBKB, CCND2, CCND1, PLAU, CASP3, PIM1, EP300, HRAS, PDGFRB, PDGFRA, ABCC1, MAP2K1, PRKCB, DNMT3A, PRKCA, MMP9, SIRT1, PIK3CA, CCNE1, EZR, RAF1, MET, TP53, HDAC4, HDAC5, HDAC2, ABCB1, ROCK1, HDAC1, PIK3R1, PTGS2, EGFR, ERBB2, ABL1, HMOX1, MAPK1, MCL1, MAPK3, CREBBP, STAT3, MTOR, VEGFA, CDK6, BCL2, MDM2, MDM4, FGFR3 |
| KEGG  PATHWAY | hsa05167 | Kaposi sarcoma-associated herpesvirus infection | 49 | 1.17E-22 | GSK3B, PIK3CD, PIK3CB, FGF2, PIK3CG, ICAM1, CASP9, IKBKB, TBK1, CASP8, CCND1, CASP3, AKT2, AKT1, EP300, RAC1, JAK2, IKBKE, HRAS, CCR4, JAK1, MAP2K1, SYK, FOS, HCK, PIK3CA, RAF1, IL6ST, TP53, SRC, PIK3R1, PTGS2, HIF1A, RELA, MAPK9, MAPK8, MAPK1, MAPK3, LYN, JUN, CREBBP, STAT1, STAT3, MAPK14, MTOR, VEGFA, IL6, CDK6, CDK4 |
| KEGG  PATHWAY | hsa05207 | Chemical carcinogenesis - receptor activation | 49 | 6.92E-21 | HSP90AB1, PIK3CD, PIK3CB, AHR, FGF2, CCND1, AKT2, AKT1, JAK2, IKBKE, HRAS, CHRNB2, MAP2K1, HSP90AA1, PRKCB, PRKCA, FOS, AR, PIK3CA, BIRC5, PGR, RAF1, PPARA, CHRNA3, SRC, CHRNA4, NR1I3, XIAP, ADRB2, PIK3R1, CYP3A4, EGFR, RELA, HSP90B1, RXRA, MAPK1, MAPK3, GSTM2, JUN, VDR, EPHX1, STAT3, ESR1, MTOR, ESR2, VEGFA, CYP1A2, CYP1A1, BCL2 |
| KEGG_PATHWAY | hsa05161 | Hepatitis B | 47 | 1.74E-24 | PCNA, SRC, PIK3CD, PIK3CB, PIK3R1, TNF, RELA, CASP9, IKBKB, MAPK9, MAPK8, CASP8, TBK1, CASP3, AKT2, AKT1, MAPK1, EP300, STAT6, JAK2, HRAS, JAK3, IKBKE, JAK1, MAPK3, JUN, MAP2K1, CREBBP, PRKCB, STAT1, STAT3, PRKCA, BRAF, FOS, MAPK14, MMP9, TGFBR1, TGFBR2, CCNA2, IL6, PIK3CA, CCNE1, CDK2, BCL2, BIRC5, RAF1, TP53 |
| KEGG  PATHWAY | hsa05165 | Human papillomavirus infection | 47 | 1.37E-11 | PTGER4, GSK3B, HDAC2, HDAC1, ITGB3, PIK3CD, PSEN1, PIK3CB, PIK3R1, PTGS2, TNF, PRKCZ, RELA, EGFR, PPP2CA, IKBKB, CDC42, CASP8, TBK1, CCND2, CCND1, TERT, CASP3, AKT2, AKT1, MAPK1, EP300, HRAS, IKBKE, JAK1, MAPK3, PDGFRB, MAP2K1, CREBBP, STAT1, MTOR, PTK2, VEGFA, CCNA2, CDK6, PIK3CA, CCNE1, CDK4, CDK2, MDM2, RAF1, TP53 |
| KEGG  PATHWAY | hsa04014 | Ras signaling pathway | 45 | 9.76E-16 | CSF1R, FLT1, FLT3, FLT4, PIK3CD, PIK3CB, PIK3R1, FGF2, RELA, EGFR, IGF1R, IKBKB, CDC42, MAPK9, PAK1, MAPK8, TBK1, AKT2, KDR, ABL1, AKT1, MAPK1, RAC1, HRAS, MAPK3, PDGFRB, NTRK1, PDGFRA, MAP2K1, PRKCB, INSR, PLA2G2A, PRKCA, PTPN11, GRIN2B, VEGFA, PIK3CA, KIT, TEK, RAF1, MET, FGFR3, FGFR1, BCL2L1, EPHA2 |
| KEGG  PATHWAY | hsa05203 | Viral carcinogenesis | 44 | 1.48E-17 | HDAC4, HDAC5, HDAC2, HDAC3, HDAC1, SRC, PIK3CD, PIK3CB, PIK3R1, HDAC8, HDAC9, RELA, HDAC6, HDAC7, CDC42, POLB, CASP8, CCND2, CCND1, CASP3, CHEK1, MAPK1, EP300, RAC1, HRAS, JAK3, CCR4, JAK1, MAPK3, LYN, JUN, CREBBP, SYK, STAT3, CCNA2, CDK6, PIK3CA, CCNE1, CDK4, CDK2, CDK1, MDM2, IL6ST, TP53 |
| KEGG  PATHWAY | hsa04015 | Rap1 signaling pathway | 44 | 4.59E-17 | CSF1R, FLT1, SRC, FLT4, ITGB3, LPAR1, FPR1, LPAR2, PIK3CD, PIK3CB, PIK3R1, FGF2, PRKCZ, EGFR, IGF1R, CDC42, AKT2, KDR, AKT1, MAPK1, RAC1, DRD2, HRAS, MAPK3, PDGFRB, PDGFRA, MAP2K1, PRKCB, INSR, F2R, PRKCA, BRAF, MAPK14, GRIN2B, VEGFA, ADORA2A, PIK3CA, KIT, TEK, RAF1, MET, FGFR3, FGFR1, EPHA2 |
| KEGG  PATHWAY | hsa05208 | Chemical carcinogenesis - reactive oxygen species | 44 | 5.72E-16 | SRC, PIK3CD, AHR, PIK3CB, PIK3R1, HIF1A, RELA, EGFR, IKBKB, MAPK9, MAPK8, AKT2, ABL1, AKT1, HMOX1, MAPK1, COX2, COX1, RAC1, HRAS, ACP1, MAPK3, PTPN1, CBR1, GSTM2, JUN, MAP2K1, PDPK1, EPHX1, AKR1C3, AKR1A1, PTPN11, BRAF, FOS, MAPK14, PTK2, VEGFA, PIK3CA, CYP1A2, CYP1A1, NOX4, RAF1, MET, NOX1 |
| KEGG  PATHWAY | hsa05163 | Human cytomegalovirus infection | 44 | 7.58E-16 | PTGER4, GSK3B, ROCK1, ROCK2, SRC, ITGB3, PTGER3, PIK3CD, PIK3CB, PIK3R1, PTGS2, TNF, RELA, EGFR, CASP9, IKBKB, CASP8, TBK1, CCND1, CASP3, AKT2, CXCR2, AKT1, MAPK1, RAC1, HRAS, JAK1, MAPK3, PDGFRA, MAP2K1, PRKCB, STAT3, PRKCA, MAPK14, MTOR, PTK2, VEGFA, IL6, CDK6, PIK3CA, CDK4, MDM2, RAF1, TP53 |
| KEGG  PATHWAY | hsa04080 | Neuroactive ligand-receptor interaction | 44 | 1.48E-08 | PTGER4, CHRM2, OPRD1, CHRM3, CHRNA3, THRB, THRA, CHRNA5, CHRNA4, C5AR1, PTGER3, LPAR1, FPR1, LPAR2, ADRA1D, PLG, GRIK2, ADRB2, FPR2, ADRA1B, NR3C1, ADRA1A, EDNRA, EDNRB, HRH2, ADORA1, KISS1R, S1PR1, BDKRB2, DRD2, CHRNB2, GPR35, F2R, TACR1, OPRM1, F2, ADRA2B, GRIN2B, HCRTR1, P2RX7, ADORA2A, CCKBR, AGTR1, F2RL1 |
| KEGG  PATHWAY | hsa05010 | Alzheimer disease | 44 | 5.66E-08 | APP, CHRM3, GSK3B, PSEN2, PIK3CD, PSEN1, PIK3CB, PIK3R1, PTGS2, TNF, RELA, CASP9, IKBKB, MAPK9, CASP7, MAPK8, CASP8, TUBB3, CASP3, AKT2, AKT1, MAPK1, COX2, COX1, NOS1, CAPN1, HRAS, MAPK3, MAP2K1, MME, NOS2, CSNK2A1, INSR, ADAM10, BRAF, GRIN2B, MTOR, IL6, PIK3CA, CDK5, NOX4, MAPT, RAF1, NOX1 |
| KEGG  PATHWAY | hsa04510 | Focal adhesion | 43 | 5.02E-17 | GSK3B, FLT1, ROCK1, ROCK2, SRC, FLT4, ITGB3, XIAP, PIK3CD, PIK3CB, PIK3R1, EGFR, IGF1R, MYLK, CDC42, MAPK9, PAK1, MAPK8, CCND2, CCND1, AKT2, ERBB2, KDR, AKT1, MAPK1, RAC1, HRAS, MAPK3, PDGFRB, PDGFRA, JUN, MAP2K1, PRKCB, PDPK1, PRKCA, BRAF, PTK2, PPP1CA, VEGFA, PIK3CA, BCL2, RAF1, MET |
| KEGG  PATHWAY | hsa05022 | Pathways of neurodegeneration - multiple diseases | 43 | 4.05E-05 | APP, CHRM3, GSK3B, VCP, PSEN2, PSEN1, PTGS2, TNF, RELA, CASP9, MAPK9, CASP7, MAPK8, CASP8, TBK1, TUBB3, CASP3, MAPK1, COX2, COX1, NOS1, RAC1, CAPN1, HRAS, MAPK3, MAP2K1, NOS2, HSPA5, CSNK2A1, PRKCB, PRKCA, BRAF, MAPK14, GRIN2B, MTOR, IL6, CDK5, BCL2, NOX4, MAPT, RAF1, NOX1, BCL2L1 |
| KEGG  PATHWAY | hsa05166 | Human T-cell leukemia virus 1 infection | 42 | 1.34E-14 | XIAP, PIK3CD, PIK3CB, PIK3R1, TNF, RELA, ICAM1, IKBKB, POLB, MAPK9, CCNB2, MAPK8, XPO1, CCND2, CCND1, TERT, CHEK2, AKT2, CHEK1, AKT1, MAPK1, EP300, HRAS, JAK3, JAK1, MAPK3, JUN, MAP2K1, CREBBP, MMP7, FOS, TGFBR1, IL2, TGFBR2, CCNA2, IL6, PIK3CA, CCNE1, CDK4, CDK2, TP53, BCL2L1 |

**Supplementary Table 6 IC50 and RI of NCI-N87 cell line at different stages of resistance to trastuzumab**

| **Resistance time** | **Resistance concentration (μg/ml)** | **IC50 (****μg/ml)** | **Resistance index (RI)** |
| --- | --- | --- | --- |
| Untreated NCI-N87 | 0 | 14.35 |  |
| trastuzumab 1 month | 88 | 35.25 | 2.46 |
| trastuzumab 2 months | 500 | 66.55 | 4.64 |
| trastuzumab 3 months | 1500 | 145.5 | 10.14 |
| trastuzumab 4 months | 3000 | 176.5 | 12.30 |
| trastuzumab 5 months | 3600 | 212.2 | 14.79 |
| trastuzumab 6 months | 3000 maintenance | 190.3 | 13.26 |
